# Monolayer Organic Crystals for Ultrahigh Performance Molecular Diodes

**DOI:** 10.1002/advs.202305100

**Published:** 2023-12-25

**Authors:** Yating Li, Jiacheng Xie, Li Sun, Junpeng Zeng, Liqi Zhou, Ziqian Hao, Lijia Pan, Jiandong Ye, Peng Wang, Yun Li, Jian‐Bin Xu, Yi Shi, Xinran Wang, Daowei He

**Affiliations:** ^1^ National Laboratory of Solid‐State Microstructures School of Electronic Science and Engineering Key Lab of Optoelectronic Devices and Systems with Extreme Performances and Collaborative Innovation Center of Advanced Microstructures Nanjing University Nanjing 210093 China; ^2^ National Laboratory of Solid‐State Microstructures Jiangsu Key Laboratory of Artificial Functional Materials College of Engineering and Applied Sciences and Collaborative Innovation Center of Advanced Microstructures Nanjing University Nanjing 210023 China; ^3^ Department of Physics University of Warwick Coventry CV4 7AL United Kingdom; ^4^ Department of Electronic Engineering and Materials Science and Technology Research Center The Chinese University of Hong Kong Hong Kong 999077 China; ^5^ School of Integrated Circuits Nanjing University Suzhou 215163 China

**Keywords:** large‐area arrays, molecular diodes, monolayer organic crystals, ultrahigh‐performance

## Abstract

Molecular diodes are of considerable interest for the increasing technical demands of device miniaturization. However, the molecular diode performance remains contact‐limited, which represents a major challenge for the advancement of rectification ratio and conductance. Here, it is demonstrated that high‐quality ultrathin organic semiconductors can be grown on several classes of metal substrates via solution‐shearing epitaxy, with a well‐controlled number of layers and monolayer single crystal over 1 mm. The crystals are atomically smooth and pinhole‐free, providing a native interface for high‐performance monolayer molecular diodes. As a result, the monolayer molecular diodes show record‐high rectification ratio up to 5 × 10^8^, ideality factor close to unity, aggressive unit conductance over 10^3^ S cm^−2^, ultrahigh breakdown electric field, excellent electrical stability, and well‐defined contact interface. Large‐area monolayer molecular diode arrays with 100% yield and excellent uniformity in the diode metrics are further fabricated. These results suggest that monolayer molecular crystals have great potential to build reliable, high‐performance molecular diodes and deeply understand their intrinsic electronic behavior.

## Graphical abstract

The low rectification ratio, low conductance, and large contact resistance severely limit the development of molecular diodes. This study utilizes solution‐shearing epitaxy to assemble large‐area few‐layer C_6_‐DNTT single crystals on metals and demonstrates asymmetry platinum (Pt) / 1L C_6_‐DNTT / titanium (Ti) molecular diodes. The devices exhibit record‐high rectification ratio, aggressive unit conductance, excellent electrical stability, and high yield.

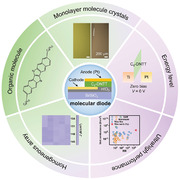

## 1 Introduction

Molecular rectification diodes operating in quantum tunneling regimes are strongly appealing for a range of next‐generation electrical circuit applications such as rectifiers, amplifiers, and energy harvesters.^[^ ^1^, ^2^, ^3^ ^]^ These applications require a high rectification ratio (RR), high conductance, and reliable electrical stability. Over the past 50 years, tremendous progress has been made to improve molecular diode performance through designing novel organic molecules,^[^ ^4^, ^5^ ^]^ developing suitable contact techniques,^[^ ^6^, ^7^, ^8^ ^]^ optimizing interfacial properties and molecular packing,^[^ ^8^, ^9^ ^]^ and adjusting energy level alignment.^[^ ^3^, ^10^ ^]^ Several mechanisms of rectifications in molecular diodes, originating from asymmetry molecule–electrode contact,^[^ ^7^, ^11^, ^12^, ^13^, ^14^, ^15^, ^16^ ^]^ multiple molecular orbitals,^[^ ^16^, ^17^ ^]^ conformation of molecule,^[^ ^18^ ^]^ and molecular dipole^[^ ^18^, ^19^ ^]^ have been fully revealed. However, so far, most molecular diodes only demonstrated typical RRs ranging from 10^2^ to 10^5^ that are three orders of magnitude lower than inorganic diodes,^[^ ^20^, ^21^ ^]^ the contact resistances in the range of 1–100 MΩ that behave more like insulators,^[^ ^1^, ^8^ ^]^ and the limited stability.^[^ ^8^, ^22^ ^]^ An efficient technique for fabricating molecular devices with high yield and integration is still lacking.

The main cause of the above challenges is the poor molecule‐electrode contact interface. In commonly used metal‐molecule‐metal break junctions, a large electric field and local heating may cause potential unexpected rearrangement or movement of atoms at the metal electrode surface during device operation,^[^ ^23^, ^24^ ^]^ which strongly impacts the diode electrical performance and stability. For the conventional deposition process of metal, the high‐energy metal ions bombarding the monomolecular film is easy to short the junction. Inserting a buffer layer between monomolecular film and metal interface favors the contact to some extent, however, the high density of disorders and traps remains due to thermal treatment,^[^ ^25^, ^26^ ^]^ leading to a large Schottky barrier regardless of materials combinations. The liquid metal allows to formation of the atomic‐level contact interface, however, it is hard to make the device miniaturization and integration.^[^ ^1^ ^]^ Therefore, it is of great significance to simultaneously realize the condensed monomolecular crystalline film and well‐defined intrinsic contact interface, which could preserve molecular intrinsic properties and improve the electrical characteristics and stability of molecular diodes.

Here, we report a controllable epitaxial growth of C_6_‐DNTT crystals down to monolayer on several classes of metal (platinum (Pt), gold (Au), and titanium (Ti)) substrates and integration of high‐performance molecular diodes by laminating top electrode of Pt. By designing the combination of contact, we observe that the rectification behavior can be tuned over eight orders of magnitude, along with high unit conductance, high breakdown electric field, and excellent current stability against bias cycling. Our results demonstrate that the monolayer small‐molecule crystals hold tremendous potential for building high‐performance diodes and as clean systems for directly characterizing device physics. Furthermore, the large‐area molecular array shows excellent variability and device yield, which potentially makes our approach scalable for circuit‐level integration.

## 2 Results

### 2.1 Growth and Characterization of Single Crystals on Metal Substrates

**Figure 1:**
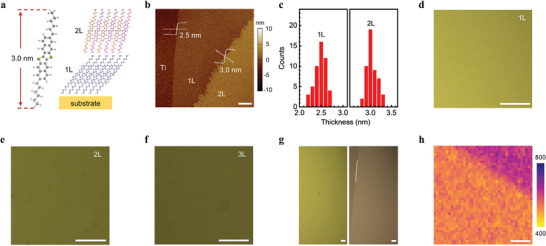
Growth of solution‐sheared C_6_‐DNTT crystals on titanium film. a) Schematic of C_6_‐DNTT molecule structure (left panel) and their packing on metal or oxide substrate (right panel). b) Atomic force microscope (AFM) image of C_6_‐DNTT crystals on Ti. The layer numbers and height profiles are marked. Scale bar, 2 µm. c) Histogram distribution of layer thickness of C_6_‐DNTT crystals on Ti, each taken from over 30 samples. The average thickness of the 1L and 2L were ≈ 2.5 and 3.0 nm, respectively. d–f) Optical micrographs of solution‐sheared C_6_‐DNTT crystals on Ti, formed with shearing speeds and growth temperatures of 2.5 µm s^−1^ and 64 °C, 2.5 µm s^−1^ and 66 °C and 2.0 µm s^−1^ and 66 °C, respectively. Scale bars, 100 µm. g) Optical micrograph (left panel) and cross‐polarized optical micrograph (right panel) of uniform 1L C_6_‐DNTT crystals on Ti. The dashed line shows the domain boundary. Scale bars, 100 µm. h) SHG mapping of 1L C_6_‐DNTT crystals from the marked area in Figure S1b (Supporting Information). Scale bar, 20 µm.

We used solution‐shearing epitaxy to deposit few‐layer C_6_‐DNTT (**Figure** **1**a) crystals on several classes of metal film (Pt, Au, and Ti) and HfO_2_ substrates by home‐built setup as shown in Figure S1a (Supporting Information). The droplet of C_6_‐DNTT saturated solution was injected into the blade‐substrate gap and dragged by the blade with a certain shearing speed to begin growing. By optimizing the growth temperature and shearing speed, the highly uniform few‐layer C_6_‐DNTT crystals were obtained (see Experimental Section for details). Below we focus our discussion on Ti substrate, while similar growth behaviors were observed on Pt, Au, and HfO_2_. As shown in Figure 1b, the crystal on Ti adopted a layer‐by‐layer packing fashion with atomic smoothness and no pinhole. The average thickness of the first layer (1L) and second layer (2L) was ≈2.5 and 3.0 nm (Figure 1c, Figure S2 in Supporting Information), respectively. The subsequent layers had the same height as 2L, indicating that molecular packing in these layers could be considered as the form of thin‐film or bulk phase of C_6_‐DNTT. The reduced height of 1L compared with 2L suggested more inclined molecular packing at the interface. Because of the weak molecule‐Ti interaction, the additional interfacial layer that molecules adopted a face‐on configuration along the substrate surface, just as pentacene and C_8_‐BTBT on graphene,^[^ ^27^, ^28^ ^]^ was not observed. We also analyzed the height of each layer C_6_‐DNTT on HfO_2_, and the results showed a similar distribution of layer thickness as C_6_‐DNTT on Ti (Figure S3, Supporting Information).

To further device applications, it is important to realize the large‐area uniform crystal as well as patterning capability. We carefully controlled the growth parameters on Ti (Figure S1, Supporting Information). The uniform few‐layer C_6_‐DNTT crystals with sub‐millimeter scale and precisely controlled thickness from 1L to trilayer (3L) were repeatedly observed (Figure 1d–f), in which the layers could be easily evaluated by color contrasts under the microscope. The single‐crystalline domain of 1L C_6_‐DNTT could continue over 2 millimeters (Figure 1g). The large‐area 1L single crystals were also observed on Pt, Au, and HfO_2_ substrates (Figure S4, Supporting Information), showing the universality of our approach. We further performed the second harmonic generation (SHG) mapping, which has proved effective in detecting domain boundaries.^[^ ^29^, ^30^ ^]^ The SHG mapping presented the uniform intensity of the whole area of the single crystal (Figure 1h, Figure S5 in Supporting Information), indicating the highly ordered single‐crystalline of C_6_‐DNTT. The successful growth of the large‐area uniform crystal is attributed to the lyophilic property (Figure S6, Supporting Information) and atomic smoothness (Figure S7, Supporting Information) of the substrates, which facilitate the C_6_‐DNTT saturated solution to spread out and crystallize on the substrates. To device integration, we also demonstrated the uniform single‐crystalline 1L C_6_‐DNTT on pre‐patterned stripe‐shaped Ti. The crystallinity was verified by a cross‐polarized optical micrograph (Figure S8, Supporting Information).

**Figure 2:**
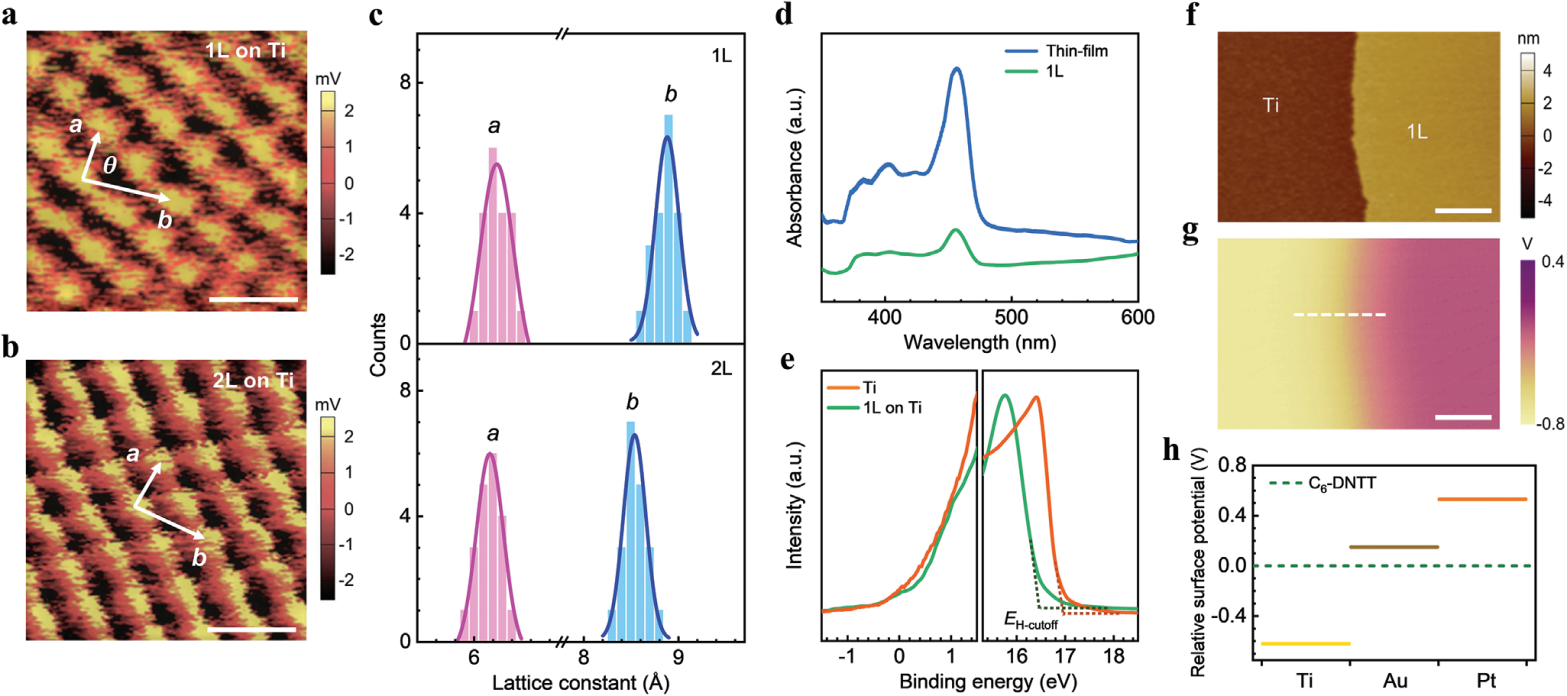
Characterization of solution‐sheared C_6_‐DNTT crystals. High‐resolution AFM images of a) 1L and b) 2L C_6_‐DNTT crystals on Ti. The unit cells are marked. Scale bars, 1 nm. All molecules in each layer adopt typical herringbone‐type packing (Figure S9 (Supporting Information) shows the high‐resolution AFM images of each layer of C_6_‐DNTT crystals on HfO_2_). c) Histogram distribution of lattice constants of 1L (upper panel) and 2L (lower panel) C_6_‐DNTT crystals on Ti, each taken from over 15 samples. Pink and blue lines show the best Gaussian fittings. d) UV–vis spectra of C_6_‐DNTT crystals on 25 nm HfO_2_/sapphire substrate. e) UPS spectra of pure Ti film and 1L C_6_‐DNTT crystal on Ti. *E*_H‐cutoff_ is the onset of secondary electron cutoff (SECO). By linear extrapolation (dashed lines), the *E*_H‐cutoff_ were calculated to be 16.45 and 16.97 eV for Ti with 1L C_6_‐DNTT and pure Ti, respectively. f) AFM topography and g) KPFM image of the same area with 1L C_6_‐DNTT crystal on Ti, respectively. Scale bars, 2 µm. h) Relative surface potentials between 1L C_6_‐DNTT crystals and different metals. The data are extracted from Figures S11, S12c,f (Supporting Information).

The high‐resolution atomic force microscope (AFM) was performed to investigate the further structural information of C_6_‐DNTT crystals (see Experimental Section for details). We found that both 1L and 2L were highly ordered structures with typical herringbone‐type packing, in which the lattice constants were *a* = 6.24 ± 0.26 Å (6.17 ± 0.27 Å), *b* = 8.88 ± 0.28 Å (8.54 ± 0.26 Å) and the angle between them was *θ* = 86° ± 1° (84° ± 3°) for 1L (2L) C_6_‐DNTT crystals on Ti (**Figure** **2**a–c). A large subnanometer‐scale error bar may be due to the thermal drift and other uncertainties such as oscillation when operating in ambient conditions. The slight expansion in 1L apparently originates from the height reduction due to substrate‐molecule interaction, which is also a major factor in inducing a slight discrepancy compared with previous reports.^[^ ^31^ ^]^ Besides substrates, the lattice strain introduced by shearing speed, which is usually used to modify the molecular packing,^[^ ^32^ ^]^ was not observed. This is because our crystals were obtained within a small shearing speed window. We also performed the high‐resolution AFM for C_6_‐DNTT crystals on HfO_2_. Compared with on Ti, the molecules followed a similar molecular packing fashion (Figure S9, Supporting Information). The observation of little statistical difference in lattice constants may come from the external perturbations.

For further insights into interfacial properties and energy level structure, we performed combined ultraviolet‐visible spectroscopy (UV–vis) and UV photoelectron spectroscopy (UPS) studies (see Experimental Section for details). The UV–vis results clearly showed that the onset of absorption peak was ≈473 and ≈ 481 nm for 1L and thin‐film C_6_‐DNTT (Figure 2d), calculating the optical bandgap of 2.62 and 2.58 eV, respectively. It closely approaches that of other DNTT‐based molecular films (C_10_‐DNTT film for ≈ 2.6 eV).^[^ ^33^, ^34^, ^35^ ^]^ Combing with UPS, the highest occupied molecular orbital (HOMO) and the lowest unoccupied molecular orbital were estimated to be −4.95 and −2.33 eV for 1L, −4.93 and −2.35 eV for thin‐film C_6_‐DNTT (Figure S10a, Supporting Information), respectively. The slight discrepancy in the energy level features may be due to the more inclined molecular packing in 1L, which has been observed in other organic small molecular systems.^[^ ^36^, ^37^ ^]^ As a comparison, the work functions of Pt and Au films were also estimated to be 5.62 and 4.96 eV (Figure S10b, Supporting Information), which are consistent with the previous reports.^[^ ^38^, ^39^, ^40^, ^41^, ^42^ ^]^ Next, we measured the UPS spectra of Ti with and without 1L C_6_‐DNTT atop it (Figure 2e). 1L C_6_‐DNTT crystal caused a remarkable ≈0.52 eV downward shift of the vacuum level, originating from interface dipoles and interfacial charge transfer, which is due to electrochemical and electrostatic effects at the interface.^[^ ^43^, ^44^ ^]^

In addition, we further proved the interfacial potential shift by kelvin probe force microscope (KPFM) measurements (see Experimental Section for details). Figure 2f,g shows AFM topography and surface potential map of the same sample. The color contrast between Ti and 1L C_6_‐DNTT clearly showed the existence of a surface potential difference, which was ≈ −0.62 V (Figure S11, Supporting Information), nearly consistent with UPS result. For 1L C_6_‐DNTT on Pt and Au, the relative surface potential differences were measured to be 0.53 and 0.15 V (Figure S12, Supporting Information), respectively. Taking the surface potential of 1L C_6_‐DNTT as zero for reference, the relative surface potentials were plotted in Figure 2h. These observations show that the relative surface potentials are not simply direct energy alignment and require careful analysis of surface dipoles. Nonetheless, these values are not accurate due to the strong effect of surface absorbents, especially in the case of ultrathin semiconductors.

### 2.2 Electrical Characterization of Molecular Diodes

**Figure 3:**
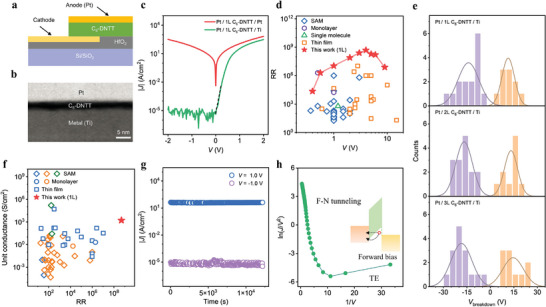
Electrical properties of molecular diodes and benchmarks. a) Schematic of monolayer molecular diode. b) The cross‐sectional STEM image of Pt / 1L C_6_‐DNTT / Ti molecular diode. c) Typical semi‐log *J–V* characteristics of Pt / 1L C_6_‐DNTT / Pt (red solid line) and Pt / 1L C_6_‐DNTT / Ti (green solid line) molecular diodes at room temperature, respectively. The black dashed line shows the ideality factor close to 1.06. d) The rectification ratio as a function of forward bias for various organic diodes. References in (d) see Table S1 (Supporting Information). e) Distribution of the forward (orange) and reverse (purple) breakdown voltages of 1L (upper panel), 2L (middle panel), and 3L (lower panel) C_6_‐DNTT molecular diodes, respectively. The corresponding **|** *J*|‐V curves see Figure S16 (Supporting Information). Each was taken from over 10 samples. For a more intuitive comparison, we conducted the Gaussian fitting for the statistical distribution of breakdown voltages. The peak of forward (reverse) breakdown voltages were located at 11.4 V (−13.8 V), 13.2 V (−16.7 V), and 14.6 V (−18.3 V) for 1L, 2L, and 3L C_6_‐DNTT molecular diodes, respectively. It implied that breakdown voltages of majority of diodes distributed around the peak. The solid lines show the Gaussian fittings. f) The unit conductance as a function of rectification ratio for various organic diodes with different contact technologies and molecules. References in (f) see Table S2 (Supporting Information). In (f), the top contacts are metal film (blue), liquid metal (orange), and STM or AFM tip (green), respectively. g) Stability of the current under continuous bias stress for Pt / 1L C_6_‐DNTT / Ti molecular diode. h) ln (*J* / *V* ^2^) ∼ V^−1^  curve of Pt / 1L C_6_‐DNTT / Ti molecular diode in (c) for the estimation of the conduction mechanism. The plot clearly shows Fowler–Nordheim tunneling at forward bias. Inset is the energy level diagram of the molecular diode at forward bias. The channel scale of all the devices in this paper is 1 µm × 2 µm unless otherwise stated.

Quantitative match with surface potential difference between 1L C_6_‐DNTT and metal (as shown in Figure 2h) allows to design monolayer molecular diode with Ohmic and Schottky contacts on both sides of a molecule, which will enhance the forward conducting current and block the reverse leakage.^[^ ^26^, ^45^ ^]^ We grew the monolayer C_6_‐DNTT crystal on stripe‐shaped metal and then laminated the pre‐patterned Pt electrode on the crystal surface (see details in Experimental Section and Figure S13, Supporting Information), forming the Pt / 1L C_6_‐DNTT / metal diode (**Figure** **3**a). The nondestructive lamination process well preserved the integrity of monolayer molecular crystal and atomically sharp contact interface, as shown by cross‐section scanning transmission electron microscopy (STEM) image of Pt / 1L C_6_‐DNTT / Ti stack (Figure 3b), which can significantly avoid Fermi level pinning, metal‐induced disorders and short.^[^ ^1^, ^46^ ^]^ Figure 3c shows the representative current density‐voltage (*J–V*) characteristics of asymmetry contact (Pt / 1L C_6_‐DNTT / Ti) and symmetry contact (Pt / 1L C_6_‐DNTT / Pt) molecular diodes, with p‐type behavior. The asymmetry molecular diode showed several remarkable features (Figure 3, Figures S14 and S15 in Supporting Information): obvious rectification behavior with rectification ratio (RR) up to 5 × 10^8^ (defined as the ratio of the forward current and the reverse current at the same |*V*|, Figure 3d and Figure S14, Supporting Information), current density ≈ 1 × 10^4^ A cm^−2^ (measured current divided by channel area, Figure S15a, Supporting Information), ideality factor close to unity (Figure 3c), reverse current as low as 10^−13^ A, turn‐on voltage below 0.3 V and reverse and forward breakdown electric field over 1×10^7^ V cm^−1^ (defined as the breakdown voltage divided by the film thickness, Figure 3e, Figure S16a,d in Supporting Information). Furthermore, thickness‐dependent conductance measurements (Figure S17, Supporting Information) showed that our diodes had a small tunneling attenuation coefficient (*β*) ≈ 0.04 Å^−1^ (the tunneling mechanism will be discussed below), which was lower than the traditional alkane^[^ ^47^ ^]^ or oligophenylene SAMs,^[^ ^48^ ^]^ and it was comparable to the ultralow *β* values of some oligomers based on ynes, porphyrins and acenes.^[^ ^49^ ^]^ The low *β* in our diodes ought to originate from atomically flat contact interface and Ohmic contact between Pt and C_6_‐DNTT.

A high breakdown electric field is important for the application of molecular electronic devices, which represents good durability and stability. Recently, several strategies, such as using more stable electrodes,^[^ ^4^ ^]^ increasing molecular length,^[^ ^50^ ^]^ and supramolecular mixing,^[^ ^51^ ^]^ have been developed to enhance the breakdown electric field of molecular junctions. Further insights into the breakdown electric field were inferred by thickness‐dependent breakdown voltage measurements for our diodes (Figure 3e and Figure S16, Supporting Information). We found that i) the breakdown voltages showed slight variation for each layer device and ii) the breakdown voltages slightly increased with the number of crystal layers increasing (Figure 3e). The former ought to come from the small discrepancy of sample induced in film and device fabrication process, and excess heat generated during device operation. The latter was mainly due to the increase of the tunneling barrier width with an increasing number of layers. Such tunneling barrier originated from the side alkyl chain of C_6_‐DNTT and molecule‐molecule interface gap, which consumed more voltage drop. The total resistance of top‐bottom electrode direct contact was 488 Ohm (Figure S18, Supporting Information), almost three orders of magnitude lower than 1L diodes, indicating that nearly all of the voltage was dropped on the molecular junction. Compared with SAMs or other non‐conjugated materials (**Table** **1**), the 1L C_6_‐DNTT bandgap is relatively small. However, the side carbon chain of C_6_‐DNTT molecule ≈ 1.4 nm is similar to SAMs or non‐conjugated materials for blocking and tunneling current. Importantly, SAMs or non‐conjugated materials assembled on metal surfaces usually have poor film quality due to the lack of pi‐pi coupling, which would generate a leakage current to reduce the breakdown electric field. Therefore, this is why our 1L C_6_‐DNTT diodes have such a high breakdown field.

**Table 1:** The comparison of rectification ratio, breakdown voltage, turn‐on voltage, and energy gap between typical previously reported organic diodes and our device.

Structure (from top to bottom)	RR	*V*_breakdown_ [V cm^−1^]	*V*_on_ [V]	Type of organic molecule	Energy gap [eV]	Top electrode	Reference
Pt/C_6_‐DNTT/Ti	4.9 × 10^8^	> 10^7^	≈0.3	Monolayer (2.5 nm)	2.6	Metal	This work
EGaIn/Ga_2_O_3_/fluorinated benzalkylsilane/Si	200	5 × 10^7^	/	SAM (0.9–1.1 nm)	/	Liquid metal	[52]
EGaIn/Ga_2_O_3_/S(CH_2_)_11_Fc/Au(Ag, Pt)	6.3 × 10^5^	±1 × 10^7^–1.5 × 10^7^	≈−1.3	SAM (2.5–2.8 nm)	2.6–2.9	Liquid metal	[4]
EGaIn/SC_11_BIPY‐ SCn/Au^TS^	100	2.45 × 10^7^	/	interstitial mixed SAM (1.9 nm)	/	Liquid metal	[53]
Au/PEDOT:PSS/pentacene/Al	20	1.2 × 10^6^(forward) 1.9 × 10^6^(reverse)	≈2.5	160 nm film	2	Metal	[54]
Au/pentacene/ZnO/ITO	3 × 10^3^	2.5 × 10^6^	1.5	100 nm film	2/3.3	Metal	[55]
Al/pentacne/Au	≈10^6^	1.6 × 10^6^(reverse)	≈1.5	160 nm film	2	Metal	[56]
Au/PQT‐12/ZnO/ITO	≈400	8*10^5^	1.2	150/30 nm film	2.27/3.3	Metal	[57]
Al/BCP/C_60_/HMDS/WO_3_/Al	4.6 × 10^4^	1.5 × 10^6^(reverse)	≈0.35	100 nm film	2.3	Metal	[58]
Al/pentacene/PFBT/Au	1.05 × 10^7^	2 × 10^6^(reverse)	/	100 nm film	2	Metal	[59]

Next, we benchmarked the RR with existing high‐performance molecular diodes using different contact techniques and different channel materials as well as morphologies, as summarized in Figure 3d and Table S1 (Supporting Information). Our diode exhibited the record high RR to date, which was one order of magnitude higher (at *V* > 1 V) than the previous reports for molecular diodes as well as organic diodes. We further summarized the breakdown electric field and unit conductance of these molecular diodes as shown in Figure 3e,f, Table 1, and Table S2 (Supporting Information). Our Pt / 1L C_6_‐DNTT / Ti device delivered the ultrahigh breakdown electric field over 1 × 10^7^ V cm^−1^ (even more than 5 × 10^7^ V cm^−1^) and unit conductance over 10^3^ S cm^−2^ while keeping high RR among all molecular diodes.^[^ ^1^ ^]^ The corresponding resistances of our devices could be down to the level of 10^−1^ MΩ (Figure 3c, Figure S14a, Supporting Information), lowering one order of magnitude to the previous reports.^[^ ^1^ ^]^ Furthermore, we conducted the continuous bias stress measurement on the Pt / 1L C_6_‐DNTT / Ti device. The result showed excellent operation stability over 1.2 × 10^4^ s with almost no degradation (Figure 3g).

The observation of superior properties for Pt / 1L C_6_‐DNTT / Ti molecular diodes requires further theoretical understanding. To this end, we first draw the energy level diagrams of Pt / 1L C_6_‐DNTT / Ti molecular diode (inset of Figure 3h and Figure S19 in Supporting Information). Since the large contact potential difference on both sides of molecule (Figure 2f–h, the Ohmic contact for Pt / 1L C_6_‐DNTT and Schottky contact for Ti / 1L C_6_‐DNTT), the triangular barrier was formed and dominated the charge transport. Under large forward bias, the shape of $\ln\frac{J}{V^{2}}∼\frac{1}{V}\;$curve showed linear decrease (Figure 3h), suggesting that the free charge carriers passed through the 1L C_6_‐DNTT by Fowler–Nordheim (F–N) tunneling.^[^ ^60^ ^]^ The tunneling effect was further confirmed by variable‐temperature measurement where the current remained unchanged at low temperatures (Figure S20, Supporting Information). For a smaller forward bias, however, no measurable current flowed through the diode prior to the onset of F‐N tunneling, since the low density of injected carriers by thermal emission would redistribute into the high density of thermally generated carriers in C_6_‐DNTT to maintain charge neutrality (Figure 3h).^[^ ^61^ ^]^ Under reverse bias, the large Schottky barrier effectively blocked the carrier injection (Figure S19, Supporting Information), creating an ultralow reverse current (Figure 3c, Figures S14 and S15 in Supporting Information). Furthermore, these phenomena were also observed in the bilayer (2L) and trilayer (3L) Pt / C_6_‐DNTT / Ti devices, where the forward current scaled down with the thickness of the C_6_‐DNTT film due to the increasing width of the tunnel barrier (Figure S17, Supporting Information). In contrast, the Pt / 1L C_6_‐DNTT / Pt and Pt / 1L C_6_‐DNTT / Au molecular diodes exhibited higher current density but nearly no rectification effect in the measured voltage range (Figure S21a, Supporting Information) due to the lack of Schottky barrier. The direct tunneling characteristics were obviously observed that the shape of $\ln\frac{J}{V^{2}}∼\frac{1}{V}\;$curves had logarithmic growth at forward bias in Pt / 1L C_6_‐DNTT / Pt and Pt / 1L C_6_‐DNTT / Au devices (Figure S21b, Supporting Information). Furthermore, we found a higher work function metal created a higher conduction current (Figure 3c and Figure S21a in Supporting Information), indicating that the sub‐HOMO also contributed to the conduction current (Figure S19, Supporting Information). Such phenomenon of current enhancement induced by sub‐HOMO levels has been widely reported in organic molecular systems.^[^ ^4^, ^17^, ^37^ ^]^ These features strongly suggest that our devices have high‐quality crystals and well‐intrinsic contact interfaces, and excellent rectification feature results from asymmetry electrical contact rather than conformation of molecule, roughness of electrode, and so on.^[^ ^3^, ^10^, ^18^ ^]^

### 2.3 Molecular Diode Array and Variability

**Figure 4:**
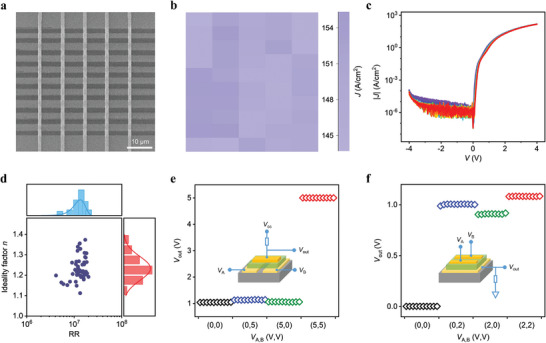
Pt / C_6_‐DNTT / Ti molecular diode array performance. a) Scanning electron micrograph image of a 5 × 10 Pt / 1L C_6_‐DNTT / Ti molecular diode array with 1.5 µm × 1.5 µm channel scale. The dark and bright stripes are Ti bottom electrodes and Pt top electrodes, respectively. b) Distribution of maximum on‐state current density and c) corresponding *J–V* characteristics of the molecular diode array. d) Statistical distribution of rectification ratios (at *V* = 3 V) and ideality factors of 50 molecular diodes. Output voltage levels of an e) AND and f) OR logic gate integrating two Pt / 1L C_6_‐DNTT / Ti molecular diodes. Insets show the circuit diagrams. The pull‐up resistor for AND gate is 1 MΩ and the pull‐down resistor for OR gate is 1 MΩ. *V*_cc_ = 5 V.

The high yield, small variation, and integration of molecular‐level devices are long‐term tremendous challenges due to the bad contact interface and poor molecular film.^[^ ^62^ ^]^ To assess the scalability of our approach, we used spin‐coated large‐area PMMA to transfer a metal array onto a monolayer C_6_‐DNTT single crystal grown on pre‐patterned Ti substrate for molecular diode array fabrication (see details in Experimental Section and Figure S22, Supporting Information). **Figure** **4**a–c showed the scanning electron microscope image, maximum current distribution, and *J–V* characteristics of a 5  ×  10 Pt / C_6_‐DNTT / Ti molecular diode array with 1.5 µm × 1.5 µm channel scale. Owing to the high‐quality single crystal film and nondisruptive contacts, we achieved 100% yield, and 3.9% on‐state current variation (deviation from the mean). Figure 4d shows the statistical analysis of ideality factors and rectification ratios of 50 molecular diodes. The average (best) ideality factor and rectification ratio were 1.24 (1.11) and 1.2 × 10^7^ (2.0 × 10^7^), respectively. Furthermore, we demonstrated logic gates by integrating two Pt / C_6_‐DNTT / Ti molecular diodes. The gates showed excellent logic function in that the high output was equivalent to the supply voltage (*V*_cc_) in AND gate and the low output was close to zero in OR gate. The non‐ideal output is because the pull‐up or pull‐down resistor consumes part of the voltage.

## 3 Discussion

In conclusion, we demonstrated the ultra‐high‐performance molecular diode by designing reasonable contact combinations. Our Pt / 1L C_6_‐DNTT / Ti molecular diode exhibited a record‐high rectification ratio of 5 × 10^8^ at *V* = 4 V, unit conductance over 10^3^ S cm^−2^ and ultrahigh breakdown electric field, as well as excellent operation stability, low turn‐on voltage, near unity ideality factor. We further demonstrated a large‐area array with 100% yield and reproducibility. These performances outperform the previously reported technologies and meet the molecular‐scale electronics. The excellent electrical performance, stability, and scalability will further advance the molecular diodes toward commercial diodes.

## 4 Experimental Section

### Preparation of Substrates

Atomic layer deposition was performed to deposit 25 nm HfO_2_ film on p‐doped silicon substrate with 285 nm SiO_2_ at 150 °C with a base pressure of ≈1 Pa, using tetrakis (dimethylamido) hafnium and H_2_O as precursors. 20 sccm N_2_ was used as carrier gas. The pulse/purge time for Hf and H_2_O precursors was 300 ms / 30 s and 30 ms / 30 s, respectively. Compared with SiO_2_, polymer or other substrates, an HfO_2_ substrate was beneficial to grow high‐quality ultrathin crystalline C_6_‐DNTT films due to high surface energy and low roughness (Figures S3–S7 in Supporting Information).^[^ ^63^ ^]^ Therefore, it benefited the growth of uniform and large‐area organic thin films on metal bottom electrodes. The stripe‐shaped metal films, including 10 nm Pt, 30 nm Au, and 30 nm Ti, were deposited on 25 nm HfO_2_ film by the standard process of electron‐beam lithography (EBL) and electron‐beam evaporation (EBE) as the bottom electrodes of molecule diodes.

### Growth of C_6_‐DNTT Crystals

The C_6_‐DNTT powder (purity over 99%, without further purification) was purchased from Lumtec, dissolved in 1,2,3,4‐tetrahydronaphthalene at 85 °C to a saturated concentration of 0.45 mg mL^−1^. The epitaxial growth of few‐layer C_6_‐DNTT crystals was performed in a home‐built solution‐shearing setup.^[^ ^64^ ^]^ The gap between the blade and the substrate was set to 100 µm. Both the blade and the substrate were set to the same growth temperature. The 20 µL droplet of C_6_‐DNTT solution was injected into the blade‐substrate gap and dragged by shearing blade across a heated metal film substrate while keeping the bulk of solution inside the blade‐substrate gap and spreading out at the front exposed region to start growth. The high surface energy and atomic smoothness of the substrate (Figures S6 and S7, Supporting Information) facilitate the solution to uniformly coat onto the metal surface and then gradually form a supersaturated zone for crystallizing large‐area crystals along with solvent evaporation. The crystallinity of solution‐sheared crystal is strongly influenced by the solute crystallization rate,^[^ ^65^, ^66^ ^]^ substrate lyophilic property, and substrate smoothness. Therefore, the selection of solvent, substrate, and solution concentration was very important to grow the ultrathin organic single crystal. Here, the growth temperature and shearing speed were finely optimized to control the thickness, morphology, and scale of C_6_‐DNTT crystals (Figure S1, Supporting Information). When increasing the growth temperature and slowing down the shearing speed, the solvent was easy to evaporate and it facilitated to formation of nucleation of 2L or thicker film on the substrate, and then assembled a complete organic thin film. This process was referred to as thermodynamic and kinetic equilibrium.^[^ ^65^, ^67^ ^]^ So, thicker film could be achieved.

### Preparation of Top Electrodes

Top electrodes were per‐patterned on silicon substrate by the standard process of EBL, and then 180 nm / 20 nm Au / Pt was evaporated by the standard process of EBE. After lift‐off, the top electrode was transferred onto C_6_‐DNTT crystals as the anode of the molecular diode under an optical microscope using a tungsten probe tip attached to a micromanipulator (Figure S13, Supporting Information).

### Characterization of C_6_‐DNTT Crystals

AFM, high‐resolution AFM, and KPFM were conducted on the Asylum Cypher S system at ambient conditions. The film thickness and topography were tested by AFM in the AC Air Topography mode. High‐resolution AFM characterized the lattice constants of C_6_‐DNTT crystals in the Lateral mode. KPFM was used to characterize the relative surface potential between C_6_‐DNTT crystals and metal films in the SKPM mode, and all samples were grounded during the KPFM scans. The scan rate in AC Air Topography mode, Lateral mode, and SKPM mode was 2, 26, and 2 Hz, respectively. SHG was performed in a customized system equipped with a piezo stage, an ultrafast laser of 1064 nm wavelength (Rainbow 1064 OEM), and a photon‐counting head (Hamamatsu H7421‐50). The cross‐polarized optical microscopy was performed by the ScanPro spectro‐microscope under white light at ambient conditions to characterize the crystallization of C_6_‐DNTT crystals. The UV–vis absorption spectra were tested with a UV–vis spectrophotometer (UV‐6100S) in 1L and thin‐film C_6_‐DNTT grown on a 25 nm HfO_2_/sapphire plate. The UPS spectra were carried out on a ThermoFisher Nexsa UV Photoemission Spectroscopy with a He I radiation source of 21.22 eV and an energy resolution <100 meV. The sample was biased −5 V to enhance the collection of electrons. The *E*_H‐cutoff_ and *E*_L‐cutoff_ could be extracted to estimate the HOMO by *E*_HOMO_ = (*h*ν − *E*_H − cutoff_)  + *E*_L − cutoff_,^[^ ^68^, ^69^ ^]^ where *E*_H‐cutoff_ and *E*_L‐cutoff_ are the onset of secondary electron cutoff (SECO) and the energy difference between the HOMO and Fermi level, and *h*ν − *E*_H − cutoff_ was corresponding Fermi level. The cross‐sectional TEM specimen was prepared using the standard FEI Scois Dualbeam focused ion beam. Then the STEM image was obtained on a FEI Titan^3^ G2 60–300 aberration corrected S/TEM at an accelerating voltage of 300 kV.

### Device Measurements

All electrical measurements of the devices were carried out by a dual‐channel SourceMeter Keithley 2636B in a closed‐cycle probe station with a base pressure of 10^−4^ Torr, which enables a current resolution of fA. In all electrical measurements, the voltage was applied to the top electrode Pt, and the bottom electrode was grounded. NPLC was set to 1. All single scans began at the negative voltage, except that the breakdown voltage measurements began at 0 V.

## Conflict of Interest

The authors declare no conflict of interest.

## Supporting information

Supporting Information

## Data Availability

Research data are not shared.
