# Supplementary material for: Monolayer Organic Crystals for Ultrahigh Performance Molecular Diodes
Source: Adv Sci (Weinh). 2023 Dec 25;11(10):2305100. doi: 10.1002/advs.202305100 (PMC10933607; doi:10.1002/advs.202305100)
Supplement: Supplementary file 1 — Supporting Information [file ADVS-11-2305100-s001.pdf]

ADVANCED SCIENCE
Open Access

Supporting Information

for *Adv. Sci.*, DOI 10.1002/advs.202305100

Monolayer Organic Crystals for Ultrahigh Performance Molecular Diodes

Yating Li, Jiacheng Xie, Li Sun, Junpeng Zeng, Liqi Zhou, Ziqian Hao, Lijia Pan, Jiandong Ye, Peng Wang, Yun Li, Jian-Bin Xu, Yi Shi*, Xinran Wang and Daowei He**

Supporting Information

Monolayer organic crystals for ultrahigh performance molecular diodes

Yating Li, Jiacheng Xie, Li Sun, Junpeng Zeng, Liqi Zhou, Ziqian Hao, Lijia Pan, Jiandong Ye, Peng Wang, Yun Li, Jian-Bin Xu, Yi Shi,* Xinran Wang and Daowei He**

Dr. Y. Li, J. Xie, L. Sun, Dr. J. Zeng, Dr. Z. Hao, Prof. L. Pan, Prof. J. Ye, Prof. Y. Li, Prof. Y. Shi, Prof. X. Wang, Prof. D. He

National Laboratory of Solid State Microstructures, School of Electronic Science and Engineering, Key Lab of Optoelectronic Devices and Systems with Extreme Performances and Collaborative Innovation Center of Advanced Microstructures, Nanjing University, Nanjing 210093, China

E-mail: hedwei@nju.edu.cn; yli@nju.edu.cn; yshi@nju.edu.cn

Dr. L. Zhou

National Laboratory of Solid State Microstructures, Jiangsu Key Laboratory of Artificial Functional Materials, College of Engineering and Applied Sciences and Collaborative Innovation Center of Advanced Microstructures, Nanjing University, Nanjing 210023, China

Prof. P. Wang

Department of Physics, University of Warwick, Coventry, CV4 7AL, UK.

Prof. J. Xu

Department of Electronic Engineering and Materials Science and Technology Research Center, The Chinese University of Hong Kong, Hong Kong 999077, China

Prof. X. Wang

School of Integrated Circuits, Nanjing University, Suzhou 215163. China

Keywords: monolayer organic crystals, molecular diodes, ultrahigh performance, large-area arrays

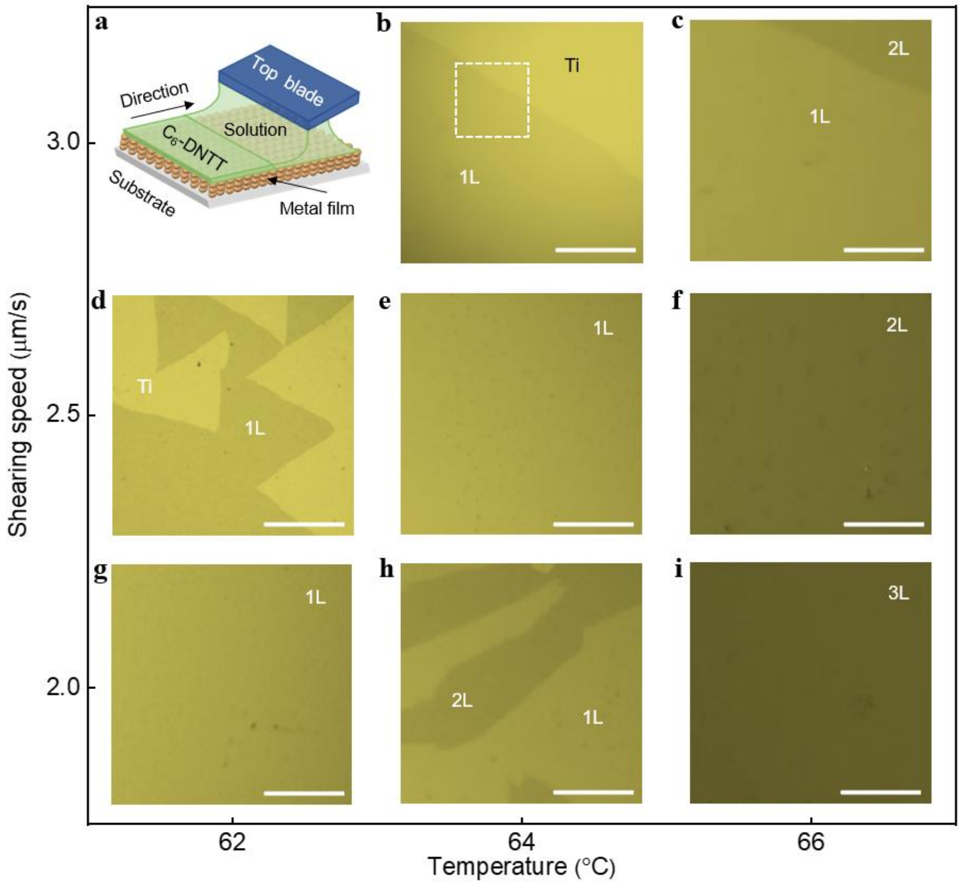

Figure S1. a) Schematic diagram of solution-sheared C_6-DNTT crystal growth method. b-i) Optical micrographs of few-layer solution-sheared C_6-DNTT crystals on Ti, formed with different shearing speeds and growth temperatures. By optimizing the growth parameters, uniform and large-area 1L, 2L and 3L C_6-DNTT crystals could be realized. Scale bars are all 100 μm. The square dashed line in (b) was performed SHG characterization as shown in Figure 1h.

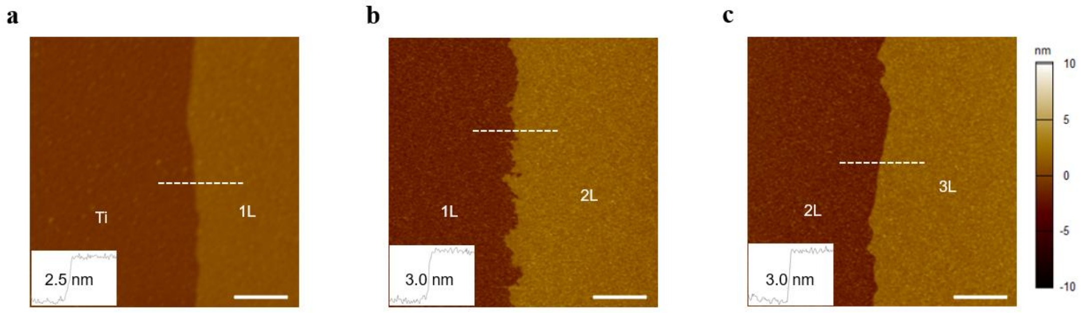

Figure S2. a-c) AFM images of each-layer C_6-DNTT crystals on Ti, respectively. The

layer numbers are marked on each image and the height profiles along the dashed lines are shown in the insets. Scale bars, 1 μm.

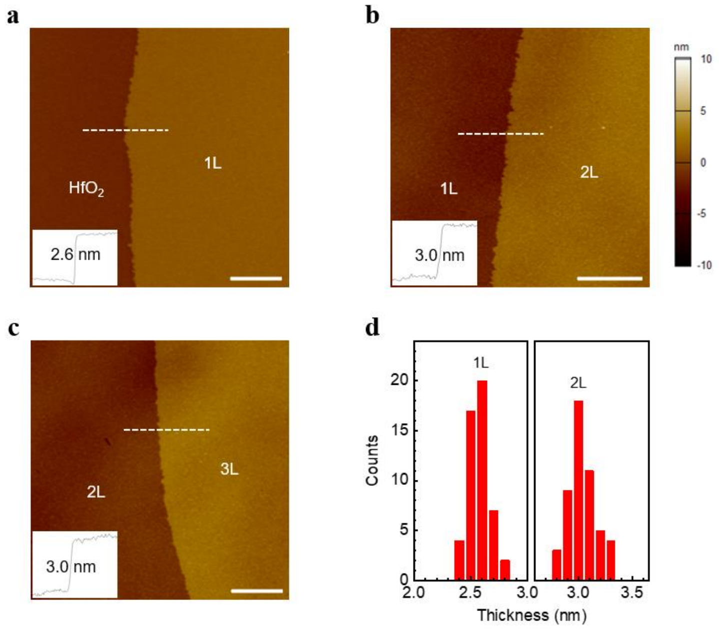

Figure S3. a-c) AFM images of each-layer C_6-DNTT crystals on HfO_2. The layer numbers are marked and the height profiles along the dashed lines are shown in the insets. Scale bars, 2 μm. d) Histogram distribution of layer thickness of C_6-DNTT crystals on HfO_2, each taken from over 30 samples. The thickness of the 1L and 2L C_6-DNTT was about 2.6 nm and 3.0 nm, respectively.

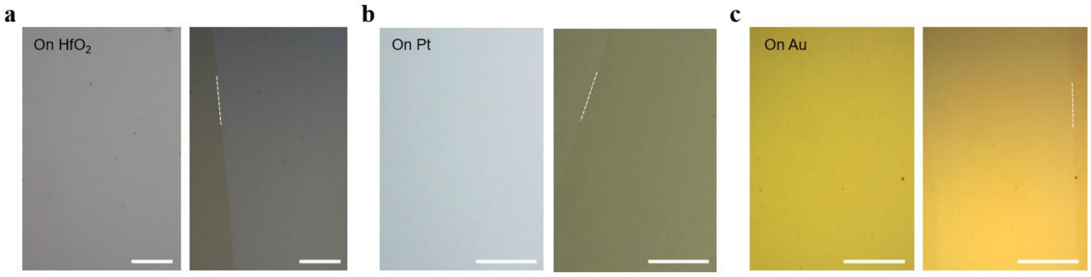

Figure S4. Optical micrographs (left panel) and cross-polarized optical micrographs (right panel) of uniform 1L C_6-DNTT crystals on a) HfO_2, b) Pt and c) Au film,

respectively. The dashed lines show the domain boundaries. Scale bars, 200 μm.

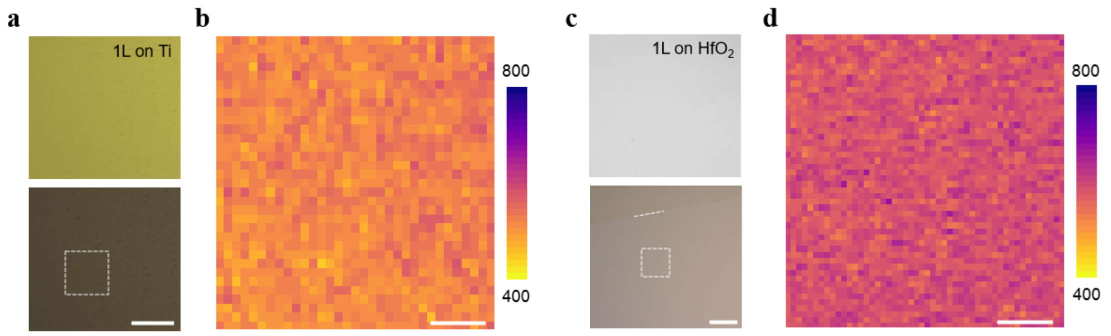

Figure S5. Optical micrographs (upper panel) and cross-polarized optical micrographs (lower panel) of uniform 1L C_6-DNTT crystals on a) Ti and c) HfO_2, respectively. The dashed line shows the domain boundary. Scale bars, 100 μm. SHG mappings of 1L C_6-DNTT on b) Ti and d) HfO_2 from the marked areas (square dashed lines) in (a) and (c), respectively. Scale bars, 20 μm.

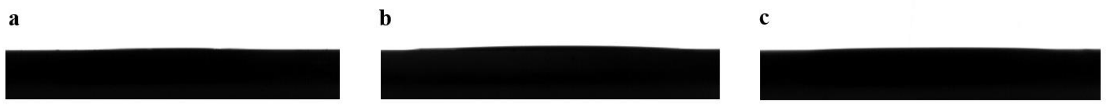

Figure S6. Contact angles of 1.9°, 3.5° and 2.4° for a droplet of tetralin on a) HfO_2, b) Ti and c) Au film, respectively. A low contact angle represents a high surface energy of the substrate, which can facilitate the C_6-DNTT solution to spread out the metal film surface.

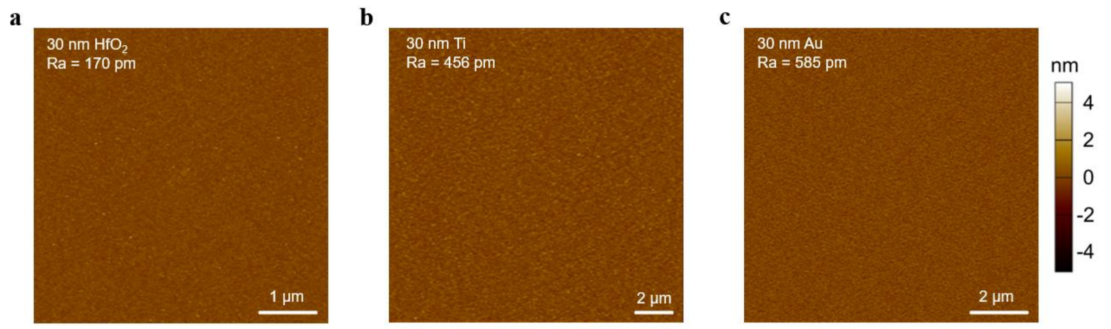

Figure S7. AFM images of a) 30 nm HfO_2, b) 30 nm Ti and c) 30 nm Au film. Ra is

the average roughness of the film surface.

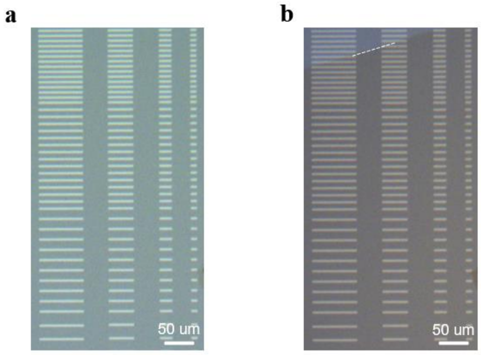

Figure S8. a) Optical micrograph and b) cross-polarized optical micrograph of uniform 1L C_6-DNTT crystals on pre-patterned stripe Ti. The dashed line in (b) shows the domain boundary.

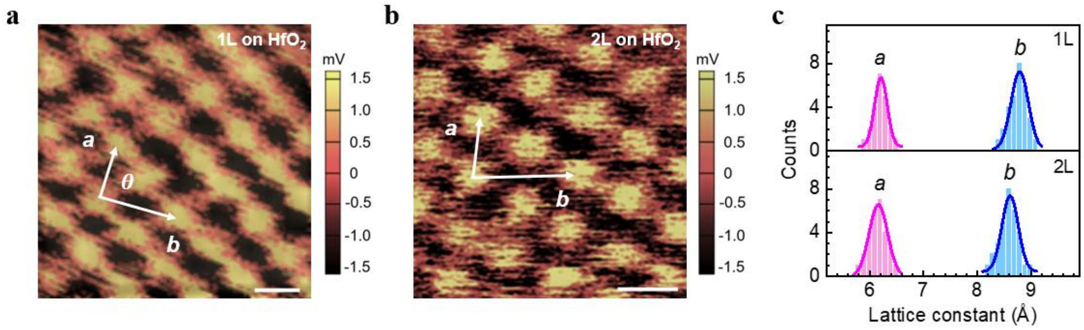

Figure S9. High-resolution AFM images of a) 1L and b) 2L C_6-DNTT crystals on HfO_2. The unit cells are marked. The scale bars, 0.5 nm. c) Histogram distribution of lattice constants of 1L (upper panel) and 2L (lower panel) C_6-DNTT crystals on HfO_2, each taken from over 15 samples. Pink and blue lines show the best Gaussian fittings. The lattice constants were a = 6.21 ± 0.31 Å (6.16 ± 0.36 Å), b = 8.79 ± 0.39 Å (8.61 ± 0.41 Å) and the angle between them was θ = 85° ± 5° (84° ± 4°) for 1L (2L) C_6-DNTT crystals on HfO_2, respectively.

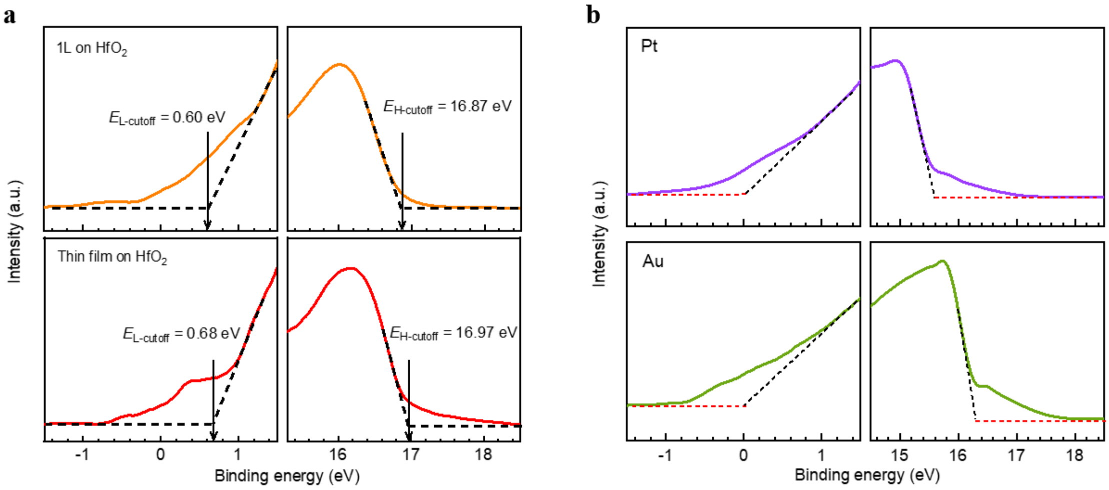

Figure S10. a) UPS spectra of 1L and thin-film C_6-DNTT crystals on 25 nm HfO_2 substrate for HOMO estimation. $E_{\text{H-cutoff}}$ and $E_{\text{L-cutoff}}$ are the onset of secondary electron cutoff (SECO) and the energy difference between the HOMO and Fermi level. By linear extrapolation (dashed lines), the $E_{\text{H-cutoff}}$ and $E_{\text{L-cutoff}}$ were calculated as 16.87 eV and 0.60 eV for 1L C_6-DNTT, 16.97 eV and 0.68 eV for thin-film C_6-DNTT, respectively. b) UPS spectra of 10 nm Pt (upper panel) and 30 nm Au (lower panel) film. The corresponding Fermi levels were estimated to be -5.62 eV and -4.96 eV, respectively.

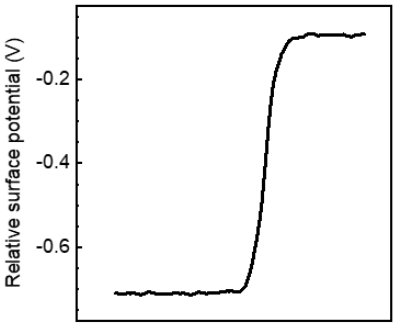

Figure S11. Relative surface potential along the dashed lines in Figure 2g. The relative potential difference between Ti and 1L C_6-DNTT crystal was about -0.62 V.

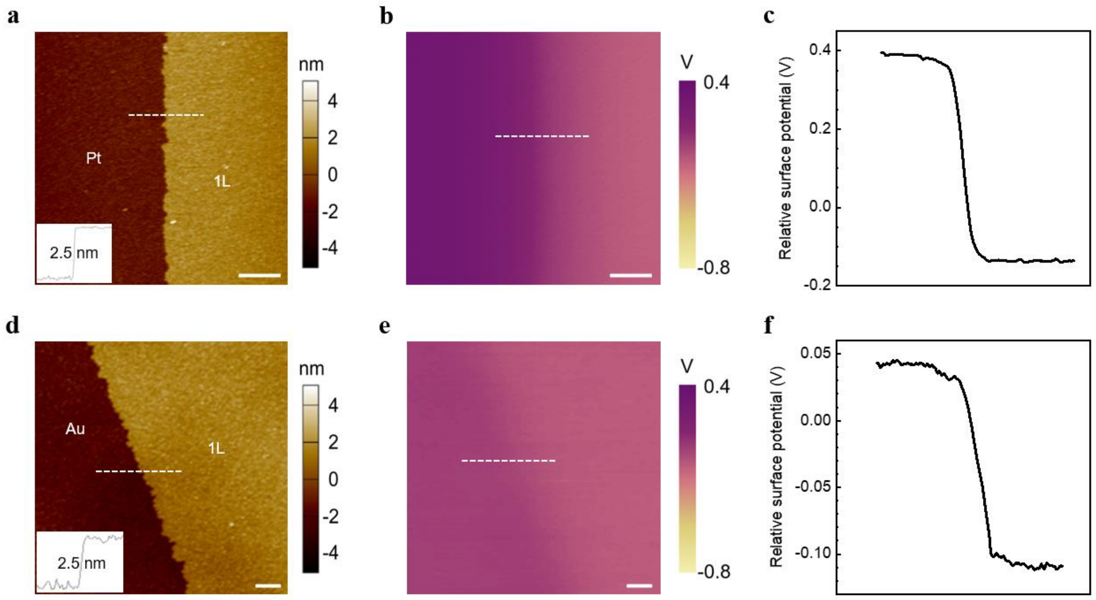

Figure S12. a, d) AFM topographies and b, e) KPFM images of the same area with 1L C_6-DNTT crystals on Pt and Au film, respectively. The layer numbers are marked and the height profiles along the dashed lines are shown in the insets in (a) and (d). Scale bars, 1 μm. c, f) Relative surface potential along the dashed lines in (b) and (e), respectively. The relative potential difference between Pt (Au) and 1L C_6-DNTT crystal was about 0.53 V (0.15 V).

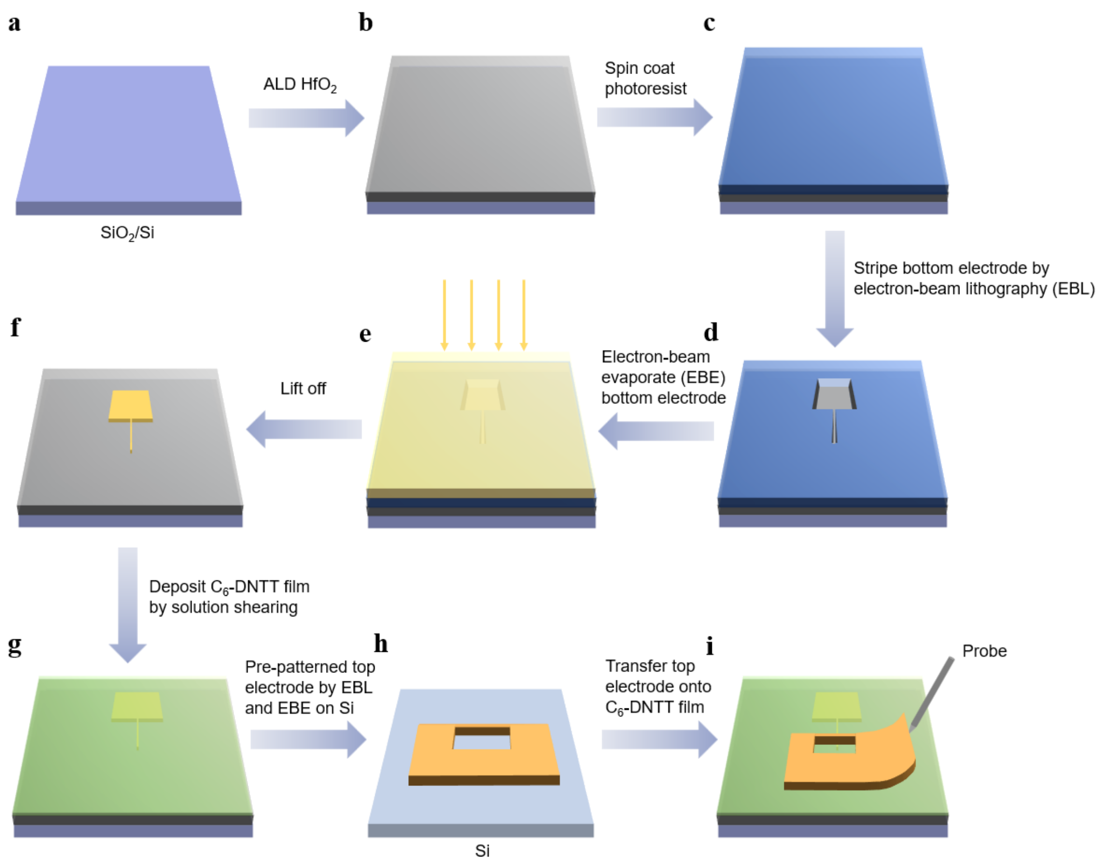

Figure S13. The fabrication process of molecular diode. a) Si substrate with 285 nm SiO_2. b) 25 nm HfO_2 was deposited on SiO_2/Si substrate by Atomic Layer Deposition (ALD). c-f) The fabrication process of bottom electrodes (including 10 nm Pt, 30 nm Au and 30 nm Ti) with the width of 1 μm by the standard semiconductor technology: spin-coated photoresist onto the surface of HfO_2/SiO_2/Si substrate (c), patterned the bottom electrodes by the standard electron-beam lithography (d), evaporated the metals by electron-beam evaporation (e) and lifted off (f). g) 1L C_6-DNTT crystal was grown onto the surface of bottom electrodes by solution shearing. h) The per-patterned 180 nm/20 nm Au / Pt top electrode with the width of 2 μm was fabricated on silicon substrate by the standard semiconductor technology. i) The top electrode was transferred onto C_6-DNTT crystals as the anode of the molecular diode. The more details of the transfer process were described in Figure S3 of Reference 1.[1]

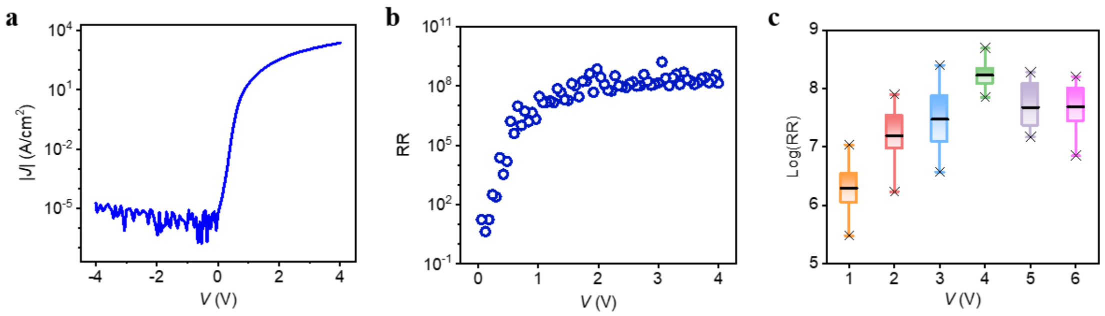

Figure S14. a) The *J*-*V* characteristic and b) rectification ratio of Pt / 1L C_6-DNTT / Ti molecular diode at room temperature. The rectification ratio was close to 1.0×10^9. c) Statistics of rectification ratio of Pt / 1L C_6-DNTT / Ti molecular diodes under different bias conditions, all taken from over 10 samples. The black lines represent the average values, the upper (lower) crosses represent the maximum (minimum) values, and the middle boxes represent 25~75 percent of the rectification ratio values.

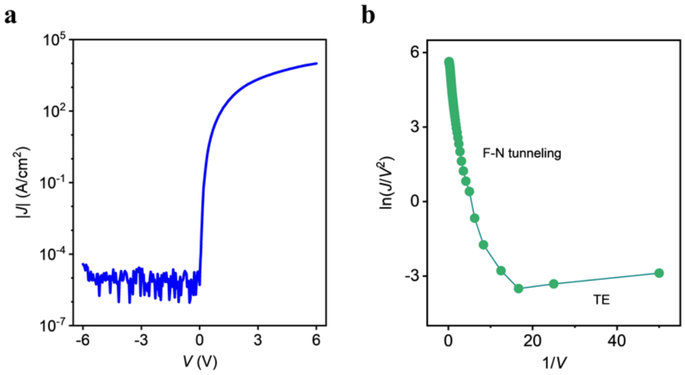

Figure S15. a) The semi-log *J*-*V* and b) ln (J/V^2)~V^{-1}. The molecular diode was measured under a larger bias condition. The plot of (b) shows the thermionic emission (TE) at small forward bias and Fowler-Nordheim (F-N) tunneling at large forward bias.

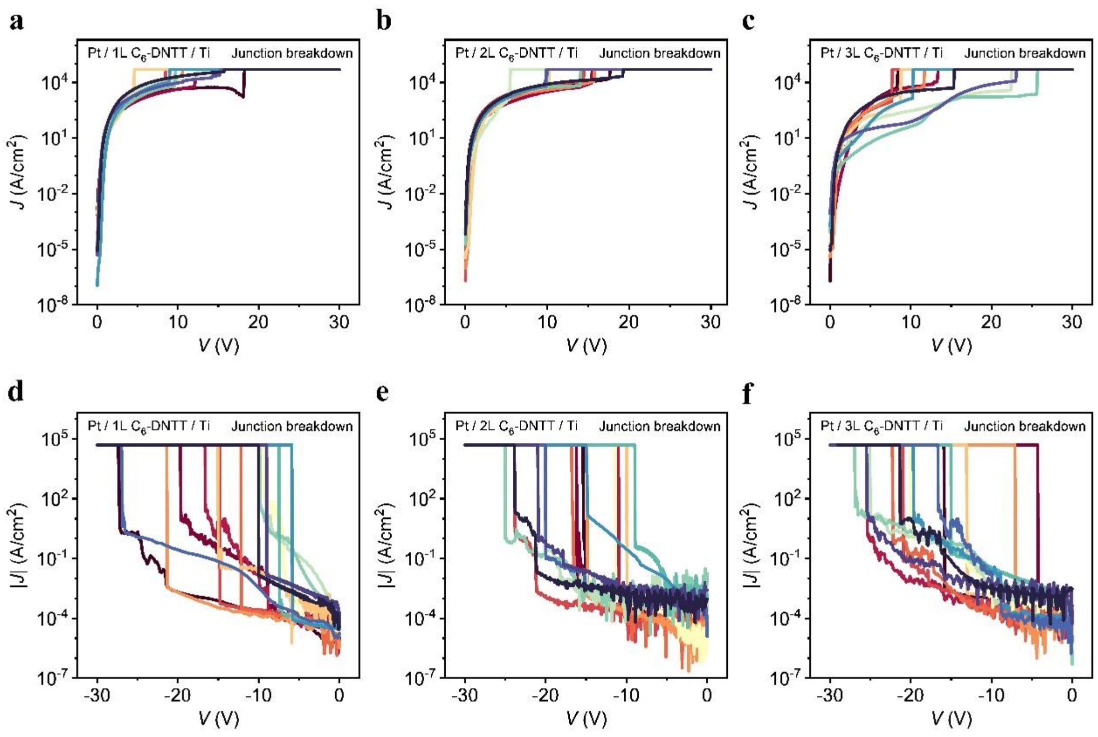

Figure S16. Forward (a ~ c) and reverse (d ~ f) breakdown voltages of 1L, 2L and 3L C_6-DNTT molecular diodes. Each taken from over 10 samples. The molecular diodes broke down when the current suddenly increased steeply. For forward bias, we observed a steady increase in current due to tunneling before breakdown (a-c). For reverse bias, we also repeatedly observed a steady increase in current due to tunneling before breakdown (d-f). On account of a larger Schottky barrier and heat effect, the reverse tunneling current of the diode had a relatively small increase before breakdown. We occasionally observed the hard breakdown of our diodes for reverse bias (d-f), which should come from the device fabrication process.

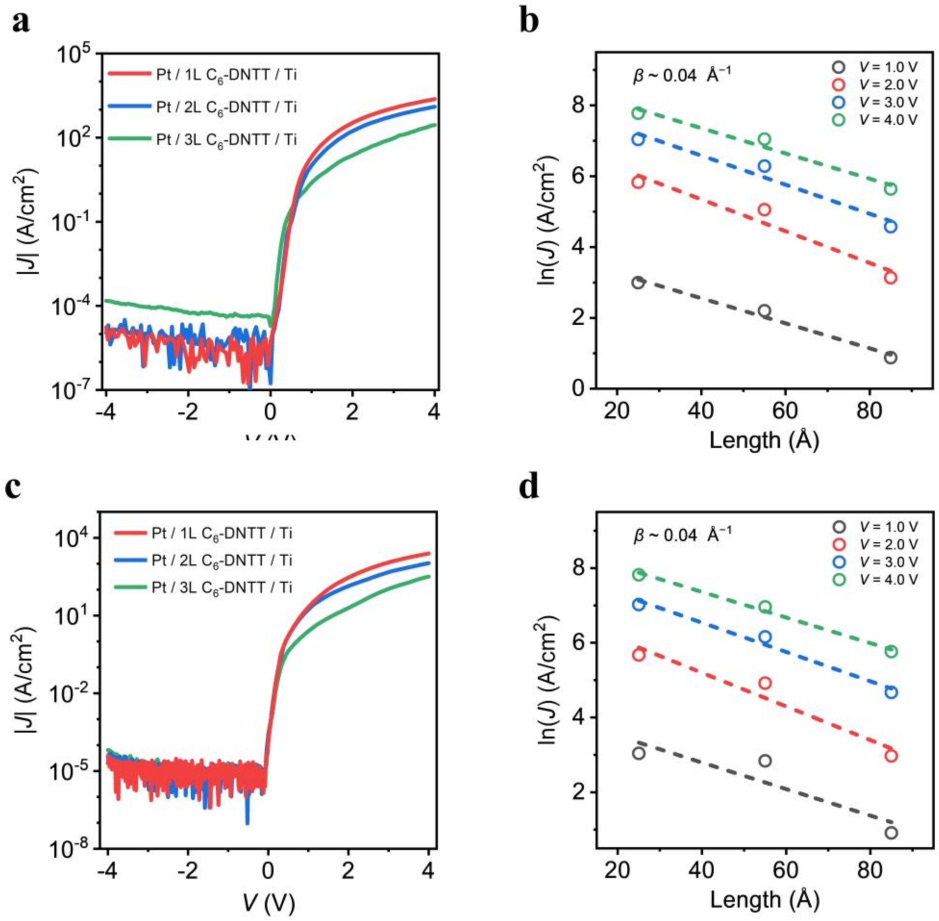

Figure S17. a) The semi-log |*J*|-*V* of Pt / C_6-DNTT / Ti molecular diodes with different C_6-DNTT layers (1L, 2L, 3L) at room temperature. b) ln (*J*)-Length curves at different applied bias. The data was extracted from **a**. The tunneling current in the molecular junction can be described as $J = J_0 e^{-\beta d}$, where *J* is the current density, J_0 is the theoretical value of *J* when $d = 0$, *β* is the tunneling attenuation coefficient and *d* is the tunneling barrier width (often approximated as the molecular length).[2, 3] From $J = J_0 e^{-\beta d}$, we can conclude that ln (*J*) is proportional to *d* and the slope of the curve is equal to –*β*. c) Another set of semi-log |*J*|-*V* curves of Pt / C_6-DNTT / Ti molecular diodes with different C_6-DNTT layers (1L, 2L, 3L) at room temperature. d) ln (*J*)-Length curves at different applied bias. The data was extracted from **c**.

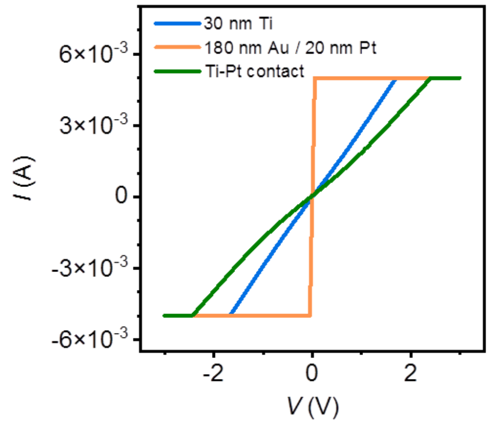

Figure S18. Conductivity measurement of the bottom electrode (blue), top electrode (orange) and the direct contact between bottom and top electrode (green). The resistance of the top (180 nm Au / 20 nm Pt) and bottom (30 nm Ti) electrodes was about 10 Ohm and 365 Ohm, respectively. The total resistance of top-bottom electrode direct contact was 488 Ohm (green), where the strip bottom electrode Ti and the strip top electrode Pt were in direct contact. From Figure 3c, Figure S14a and Figure S15a in Supporting Information, the resistance of 1L C_6-DNTT diode was about 10^{-1} MΩ ~ $5*10^{-1}$ MΩ, almost three orders of magnitude higher than the total resistance of top-bottom electrode direct contact, indicating that nearly all of the voltage was dropped on the molecular junction.

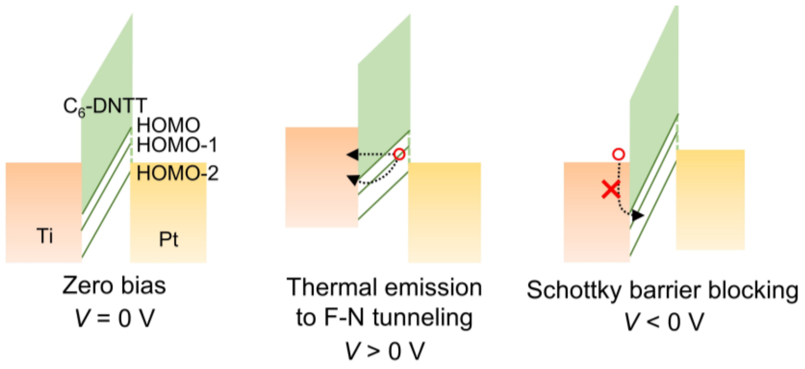

Figure S19. Energy level diagrams at zero-bias (left), forward bias (middle) and reverse bias (right) of Pt / 1L C_6-DNTT / Ti molecular diode, respectively.

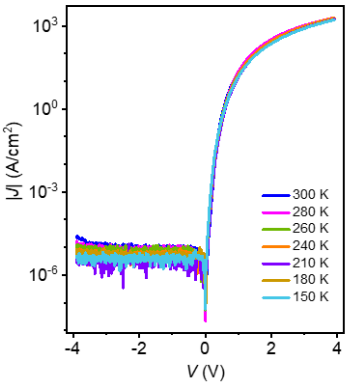

Figure S20. The variable temperature measurement of Pt / 1L C_6-DNTT / Ti molecular diode.

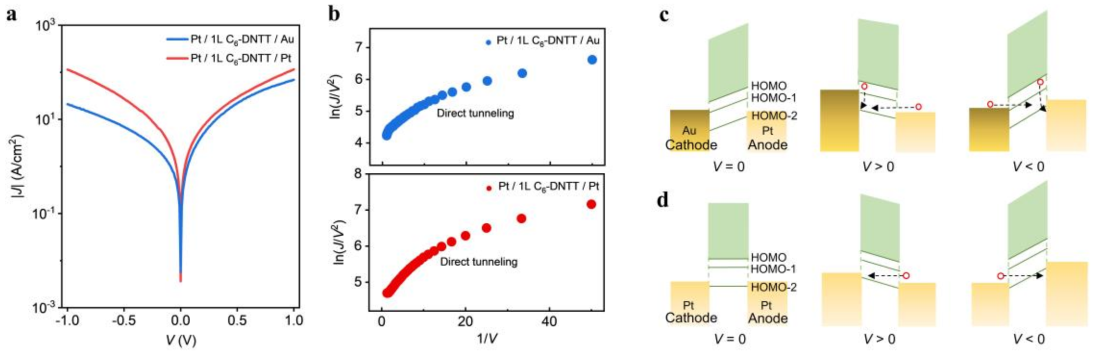

Figure S21. a) Typical semi-log *J*-*V* characteristics of Pt / 1L C_6-DNTT / Pt (red solid line) and Pt / 1L C_6-DNTT / Au (blue solid line) molecular diodes at room temperature, respectively. The Pt / 1L C_6-DNTT / Pt molecular diode is the same device in Figure 3c. b) The corresponding $\ln(J/V^2) \sim V^{-1}$ curves clearly show the direct tunneling of charge carrier injection in Pt / 1L C_6-DNTT / Pt (lower panel) and Pt / 1L C_6-DNTT / Au (upper panel) molecular diodes. Sketch of the corresponding energy level diagrams of c) Pt / C_6-DNTT / Au and d) Pt / C_6-DNTT / Pt molecular diodes under different bias conditions.

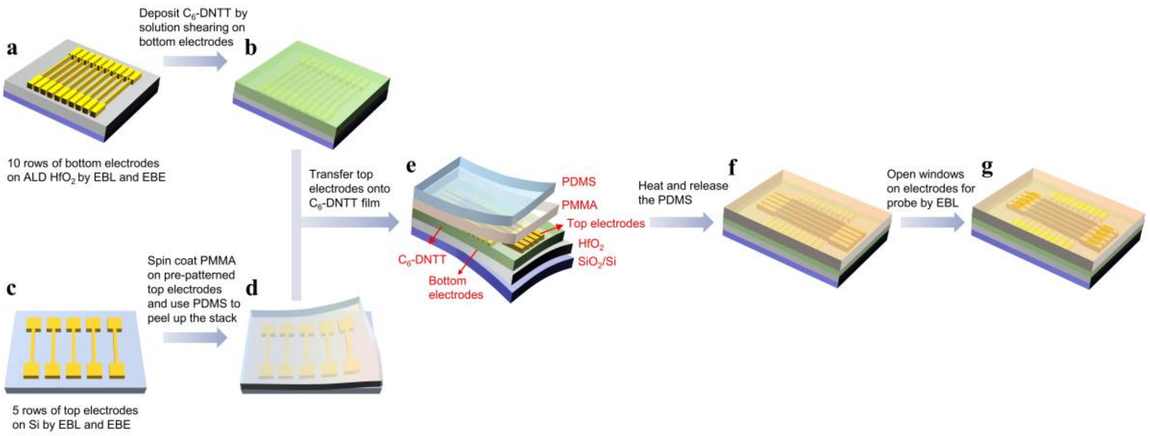

Figure S22. The fabrication process of Pt / 1L C_6-DNTT / Ti molecular diode array. a, c) The pre-patterned bottom and top electrode array were fabricated on HfO_2/Si and Si substrate by the standard semiconductor technology, respectively. The bottom electrodes are Ti, with the thickness of 30 nm and the width of 1.5 μm. The top electrodes are 180 nm/20 nm Au / Pt with the width of 1.5 μm. b) 1L C_6-DNTT crystals were grown on the surface of per-patterned Ti bottom electrodes. d) Spin-coated PMMA onto the surface of per-patterned top electrode array and the stack was peeled up by PDMS which then fixed on a glass slide. e) The PDMS / PMMA / Au / Pt stack was carefully transferred onto the surface of C_6-DNTT crystals by the transfer platform setup which can adjust temperature, height and angle. f) The PDMS was released at 90°C for 10 minutes with the heating rate of 0.02°C min^{-1} from room temperature to 90°C. g) Opened windows on the bottom and top electrodes by the standard process of electron beam lithography (EBL) to probe for electrical measurement.

Table S1. The comparison of rectification ratio under different bias conditions between typical previously reported organic diodes and our devices (the data are used in Figure 3d).

**Structure (from top to bottom)**	***V* (V)**	**RR**	**Type of organic molecule**	**Top electrode**	**Ref.**
Pt/C_6-DNTT/Ti	0.4	$2*10^4$	Monolayer	Metal	This work
Pt/C_6-DNTT/Ti	0.56	$2*10^6$	Monolayer	Metal	This work
Pt/C_6-DNTT/Ti	1	10^7	Monolayer	Metal	This work
Pt/C_6-DNTT/Ti	2	$7.9*10^7$	Monolayer	Metal	This work
Pt/C_6-DNTT/Ti	3	$2.5*10^8$	Monolayer	Metal	This work
Pt/C_6-DNTT/Ti	4	$4.9*10^8$	Monolayer	Metal	This work
Pt/C_6-DNTT/Ti	5	$1.9*10^8$	Monolayer	Metal	This work
Pt/C_6-DNTT/Ti	6	$1.6*10^8$	Monolayer	Metal	This work
Pt/C_6-DNTT/Ti	9	$7.7*10^6$	Monolayer	Metal	This work
Al/OETS-thiophene/Si	1	37	SAM	Metal	[4]
Al/OETS-thiophene(4T)/Si	1	26	SAM	Metal	[5]
Au/n-alkyl/Si	0.5	$2*10^6$	Monolayer	Metal	[6]
Au/DPE-2F/Al/Al_2O_3/ polyimide/phosporbronze	~1.2	600	Single molecule	Metal	[7]
Au/Ti/BTB/Au	2.7	$3*10^3$	9 nm film	Metal	[8]
Au/CuPc/F_{16}CoPc/Au	2	300	7 nm film	Metal	[9]
STM tip/$TDO_{n/}$Au	0.37	200	SAM	STM tip	[10]
Ag AFM tip/HSC_{11}Fc/Pt	1.5	170	SAM	AFM tip	[11]
Hg/$C_{10}H_{21}$/Si	1	10^6	SAM	Liquid metal	[12]
EGaIn/Ga_2O_3/SC_{11}Fc/Ag	1	200	SAM	Liquid metal	[13]

EGaIn/Ga_2O_3/$SC_{11}Fc_2$/Ag	1	100	SAM	Liquid metal	[14]
EGaIn/Ga_2O_3/$SC_{11}Fc$/Ag	1	180	SAM	Liquid metal	[15]
EGaIn/Ga_2O_3/SC_nFc/Ag	1	151	SAM	Liquid metal	[16]
EGaIn/Ga_2O_3/SC_{11}BIPY/Ag	1	85	SAM	Liquid metal	[17]
EGaIn/Ga_2O_3/$FcC_{11}SH$/Ag	1	98	SAM	Liquid metal	[18]
EGaIn/Ga_2O_3/$SC_{11}Fc_2$/Ag	0.875	$1.1*10^3$	SAM	Liquid metal	[19]
EGaIn/Ga_2O_3/SC_nFcC_{13-n}/Ag	1	85	SAM	Liquid metal	[20]
EGaIn/Ga_2O_3/$S(CH_2)_{13}CH_3$/Ag	1	~50	SAM	Liquid metal	[21]
EGaIn/Ga_2O_3/$S(CH_2)_{11}Fc$/Ag	0.875	$1.1*10^3$	SAM	Liquid metal	[22]
EGaIn/Ga_2O_3/$SC_{10}XFc$/Ag	1	>100	SAM	Liquid metal	[23]
EGaIn/Ga_2O_3/SC_{11}BIPY/Ag	1	85	SAM	Liquid metal	[24]
EGaIn/Ga_2O_3/HSC_{11}BIPY-HSC_X/Ag	1	131	SAM	Liquid metal	[25]
EGaIn/Ga_2O_3/fluorinated benzalkylsilane/Si	2	200	SAM	Liquid metal	[26]
EGaIn/Ga_2O_3/$S(CH_2)_{11}Fc$/Au(Ag, Pt)	3	$6.3*10^5$	SAM	Liquid metal	[27]
EGaIn/Ga_2O_3/FSC_{11}/Ag	0.5	617	SAM	Liquid metal	[28]
EGaIn/Ga_2O_3/DS-Fc-X/Au	2	99	SAM	Liquid metal	[29]
Hg/PhC/Si	1	$1.7*10^4$	Monolayer	Liquid metal	[30]
EGaIn/Ga_2O_3/ferrocenealkylsilanes/Si	1	150	SAM	Liquid metal	[31]
EGaIn/GaO_X/FcC11X/graphene/Cu	1.5	40	SAM	Liquid metal	[32]
EGaIn/Ga_2O_3/HSC_{11}BIPY-HSC_X/Ag	1	126	SAM	Liquid metal	[33]
EGaIn/$S(CH_2)_nCO_2H$/Au	1.5	316	SAM	Liquid metal	[2]

EGaIn/Ga_2O_3/S$(CH_2)_n$R$(CH_2)_m$$CH_3$/Ag	1	18.1	SAM	Liquid metal	[34]
EGaIn/Ga_2O_3/SC_{11}PAH/Ag	0.74	170	SAM	Liquid metal	[35]
EGaIn/Ga_2O_3/CPh-TPI/Si	2	2635	SAM	Liquid metal	[36]
EGaIn/Ga_2O_3/S$(CH_2)_{11}$$MV^{2+}X^{-}_2$/Ag	1	$2.5*10^4$	SAM	Liquid metal	[37]
EGaIn/Ga_2O_3/PTEG-1/Au	1	~40	SAM	Liquid metal	[38]
EGaIn/Ga_2O_3/SC_{11}BIPY/Au	1	141	SAM	Liquid metal	[39]
EGaIn/Ga_2O_3/S(CH2)$_{11}$BIPY-MCl_2/Au	1	82	SAM	Liquid metal	[40]
Hg/C_{10}SH/Si	1	$2*10^3$	SAM	Liquid metal	[41]
EGaIn/FcTEG/PTEG-1/Au^{TS}	1	316	SAB(bilayer)	Liquid metal	[42]
EGaIn/SC_{11}BIPY- SCn/Au^{TS}	1	100	interstitial mixed SAM (imSAM)	Liquid metal	[43]
AgNWs:T-TPU/SEBS/Au/PEDOT:PSS:IONE/DPP4T-oSi10/PEDOT:PSS:PSSNa:IONE/PEIE/Ag/AgNWs:T-TPU/SEBS)	2	>100	70 nm film	Metal	[44]
Al/C_{60}/Cu	2.4	$2.6*10^6$	100 nm film	Metal	[45]
Au/PEDOT:PSS/pentacene/Al	~15	20	160 nm film	Metal	[46]
Au/CuPc/Al	5	$6*10^3$	50 nm film	Metal	[47]
Au/pentacene/ZnO/ITO	5.5	$3*10^3$	100 nm film	Metal	[48]
Al/Pentacne/Au	~4.5	$\sim10^6$	160 nm film	Metal	[49]
Au/PQT-12/ZnO/ITO	2.5	~400	150 nm film	Metal	[50]
Al/BCP/C_{60}/HMDS/WO_3/Al	1	$4.6*10^4$	100 nm film	Metal	[51]

Au/pentacene/Al	10	~10^5	100 nm film	Metal	[52]
Al/pentacene/PFBT/Au	2.8	$1.05*10^7$	100 nm film	Metal	[53]
Ag/P(NDI2OD-T2)/PEI/Ag	5	10^6	200-700 nm film	Metal	[54]
Al/P3HT/PFBT/Au/Ti	4	10^6	110 nm film	Metal	[55]
Au/Cr/PgBT(F)2Gtt(p(g2T-TT), P(NDIMTEG-T))/parylene-C/Au/Cr	0.6	314.3	250-500 nm film	Metal	[56]
Ag/PEDOT:Tos/PEDOT:Tos-TDAE/Ag	5	30	260 nm film (Lateral diode)	Metal	[57]
Au/C_{60}:polystyrene/Al	2.5	10^3	700 nm film (Lateral diode)	Metal	[58]
Cu foil/T2-DPPT(DPPTTT, T-DPPT)/Cu foil	5	$2.1*10^3$	~3.3-4.5 μm film (Lateral diode)	Metal	[59]

Table S2. The comparison of rectification ratio and unit conductance between typical previously reported organic diodes and values of our devices (the data are used in Figure 3f).

**Structure (from top to bottom)**	**RR**	**Unit conductance ($S\ cm^{-2}$)**	**Type of organic molecule**	**Top electrode**	**Ref.**
Pt/C_6-DNTT/Ti	$4.9*10^8$	$1.6*10^3$	Monolayer	Metal	This work
Al/OETS-thiophene/Si	37	$9*10^{-5}$	SAM	Metal	[4]
Al/OETS-thiophene(4T)/Si	26	$9.4*10^{-3}$	SAM	Metal	[5]
Au/n-alkyl/Si	$2*10^6$	2	Monolayer	Metal	[6]
Au/Ti/BTB/Au	$3*10^3$	0.926	9 nm film	Metal	[8]

Au/CuPc/F_{16}CoPc/Au	300	157.5	7 nm film	Metal	[9]
STM tip/$TDO_{n/}$Au	200	27	SAM	STM tip	[10]
Ag AFM tip/HSC_{11}Fc/Pt	170	$1.6*10^5$	SAM	AFM tip	[11]
Hg/$C_{10}H_{21}$/Si	10^6	0.1	SAM	Liquid metal	[12]
EGaIn/Ga_2O_3/SC_{11}Fc/Ag	200	$5*10^{-3}$	SAM	Liquid metal	[13]
EGaIn/Ga_2O_3/$SC_{11}Fc_2$/Ag	100	$8*10^{-3}$	SAM	Liquid metal	[14]
EGaIn/Ga_2O_3/SC_{11}Fc/Ag	180	$1*10^{-3}$	SAM	Liquid metal	[15]
EGaIn/Ga_2O_3/SC_nFc/Ag	151	$5*10^{-4}$	SAM	Liquid metal	[16]
EGaIn/Ga_2O_3/SC_{11}BIPY/Ag	85	0.09	SAM	Liquid metal	[17]
EGaIn/Ga_2O_3/FcC_{11}SH/Ag	98	$8*10^{-3}$	SAM	Liquid metal	[18]
EGaIn/Ga_2O_3/$SC_{11}Fc_2$/Ag	$1.1*10^3$	$5*10^{-3}$	SAM	Liquid metal	[19]
EGaIn/Ga_2O_3/SC_nFcC_{13-n}/Ag	85	$1.2*10^{-4}$	SAM	Liquid metal	[20]
EGaIn/Ga_2O_3/$S(CH_2)_{13}CH_3$/Ag	~50	$8*10^{-3}$	SAM	Liquid metal	[21]
EGaIn/Ga_2O_3/SC_{10}XFc/Ag	>100	$1*10^{-3}$	SAM	Liquid metal	[23]
EGaIn/Ga_2O_3/SC_{11}BIPY/Ag	85	4.6	SAM	Liquid metal	[24]
EGaIn/Ga_2O_3/HSC_{11}BIPY-HSC_X/Ag	131	12.6	SAM	Liquid metal	[25]
EGaIn/Ga_2O_3/fluorinated benzalkylsilane/Si	200	$6*10^{-3}$	SAM	Liquid metal	[26]
EGaIn/Ga_2O_3/$S(CH_2)_{11}$Fc/Au(Ag, Pt)	$6.3*10^5$	$8*10^{-3}$	SAM	Liquid metal	[27]
EGaIn/Ga_2O_3/FSC_{11}/Ag	617	0.04	SAM	Liquid metal	[28]
EGaIn/Ga_2O_3/DS-Fc-X/Au	99	0.5	SAM	Liquid metal	[29]
Hg/PhC/Si	$1.7*10^4$	0.625	Monolayer	Liquid metal	[30]

EGaIn/Ga_2O_3/ferrocenealkylsilanes/Si	150	$4.6*10^{-5}$	SAM	Liquid metal	[31]
EGaIn/GaO_X/FcC11X/graphene/Cu	40	$4.2*10^{-4}$	SAM	Liquid metal	[32]
EGaIn/Ga_2O_3/HSC_{11}BIPY-HSC_X/Ag	126	16	SAM	Liquid metal	[33]
EGaIn/$S(CH_2)_nCO_2H$/Au	316	0.08	SAM	Liquid metal	[2]
EGaIn/Ga_2O_3/$S(CH_2)_nR(CH_2)_mCH_3$/Ag	18.1	1.6	SAM	Liquid metal	[34]
EGaIn/Ga_2O_3/SC_{11}PAH/Ag	170	0.8	SAM	Liquid metal	[35]
EGaIn/Ga_2O_3/CPh-TPI/Si	2635	$1.8*10^{-3}$	SAM	Liquid metal	[36]
EGaIn/Ga_2O_3/$S(CH_2)_{11}MV^{2+}X^{-}_2$/Ag	$2.5*10^4$	0.03	SAM	Liquid metal	[37]
EGaIn/Ga_2O_3/PTEG-1/Au	~40	0.01	SAM	Liquid metal	[38]
EGaIn/Ga_2O_3/SC_{11}BIPY/Au	141	8.5	SAM	Liquid metal	[39]
EGaIn/Ga_2O_3/$S(CH2)_{11}$BIPY-MCl_2/Au	82	1.2	SAM	Liquid metal	[40]
Hg/C_{10}SH/Si	$2*10^3$	13	SAM	Liquid metal	[41]
AgNWs:T-TPU/SEBS/Au/PEDOT:PSS:IONE/DPP4T-oSi10/PEDOT:PSS:PSSNa:IONE/PEIE/Ag/AgNWs:T-TPU/SEBS)	>100	0.935	70 nm film	Metal	[44]
Al/C_{60}/Cu	$2.6*10^6$	151.25	100 nm film	Metal	[45]
Au/PEDOT:PSS/pentacene/Al	20	140	160 nm film	Metal	[46]
Au/CuPc/Al	$6*10^3$	5.86	50 nm film	Metal	[47]
Au/pentacene/ZnO/ITO	$3*10^3$	29	100 nm film	Metal	[48]
Al/Pentacne/Au	$\sim10^6$	3.3	160 nm film	Metal	[49]
Au/PQT-12/ZnO/ITO	~400	160	150 nm film	Metal	[50]

Al/BCP/C_{60}/HMDS/WO_3/Al	$4.6*10^4$	64	100 nm film	Metal	[51]
Au/pentacene/Al	$\sim10^5$	10	100 nm film	Metal	[52]
Al/pentacene/PFBT/Au	$1.05*10^7$	33	100 nm film	Metal	[53]
Ag/P(NDI2OD-T2)/PEI/Ag	10^6	0.06	200-700 nm film	Metal	[54]
Al/P3HT/PFBT/Au/Ti	10^6	0.025	110 nm film	Metal	[55]
Au/Cr/PgBT(F)2Gtt(p(g2T-TT), P(NDIMTEG-T))/parylene-C/Au/Cr	314.3	$5*10^4$	250-500 nm film	Metal	[56]
Ag/PEDOT:Tos/PEDOT:Tos-TDAE/Ag	30	6	260 nm film (Lateral diode)	Metal	[57]
Cu foil/T2-DPPT(DPPTTT, T-DPPT)/Cu foil	$2.1*10^3$	0.766	~3.3-4.5 μm film (Lateral diode)	Metal	[59]

References

[1] D. W. He, Y. M. Pan, H. Y. Nan, S. A. Gu, Z. Y. Yang, B. Wu, X. G. Luo, B. C. Xu, Y. H. Zhang, Y. Li, Z. H. Ni, B. G. Wang, J. Zhu, Y. Chai, Y. Shi, X. R. Wang, *Appl. Phys. Lett.* **2015**, 107, 183103.

[2] Y. Ai, A. Kovalchuk, X. K. Qiu, Y. X. Zhang, S. Kumar, X. T. Wang, M. Kuhnel, K. Norgaard, R. C. Chiechi, *Nano Lett.* **2018**, 18, 7552.

[3] H. J. Lee, S. J. Cho, H. Kang, X. He, H. J. Yoon, *Small* **2021**, 17, 2005711.

[4] S. Lenfant, C. Krzeminski, C. Delerue, G. Allan, D. Vuillaume, *Nano Lett.* **2003**, 3, 741.

[5] S. Lenfant, D. Guerin, F. Tran Van, C. Chevrot, S. Palacin, J. P. Bourgoin, O. Bouloussa, F. Rondelez, D. Vuillaume, *J. Phys. Chem. B* **2006**, 110, 13947.

[6] M. A. Kuikka, W. J. Li, K. L. Kavanagh, H. Z. Yu, *J. Phys. Chem. C* **2008**, 112,

9081.
[7] M. L. Perrin, E. Galan, R. Eelkema, J. M. Thijssen, F. Grozema, H. S. van der Zant, *Nanoscale* **2016**, 8, 8919.
[8] Q. V. Nguyen, P. Martin, D. Frath, M. L. Della Rocca, F. Lafolet, C. Barraud, P. Lafarge, V. Mukundan, D. James, R. L. McCreery, J. C. Lacroix, *J. Am. Chem. Soc.* **2017**, 139, 11913.
[9] T. M. Li, V. K. Bandari, M. Hantusch, J. H. Xin, R. Kuhrt, R. Ravishankar, L. Q. Xu, J. D. Zhang, M. Knupfer, F. Zhu, D. H. Yan, O. G. Schmidt, *Nat. Commun.* **2020**, 11, 1.
[10] B. Capozzi, J. Xia, O. Adak, E. J. Dell, Z. F. Liu, J. C. Taylor, J. B. Neaton, L. M. Campos, L. Venkataraman, *Nat. Nanotechnol.* **2015**, 10, 522.
[11] Q. Van Nguyen, *J. Phys. Chem. C* **2022**, 126, 6405.
[12] Y. J. Liu, H. Z. Yu, *ChemPhysChem* **2002**, 3, 799.
[13] C. A. Nijhuis, W. F. Reus, G. M. Whitesides, *J. Am. Chem. Soc.* **2009**, 131, 17814.
[14] C. A. Nijhuis, W. F. Reus, G. M. Whitesides, *J. Am. Chem. Soc.* **2010**, 132, 18386.
[15] C. A. Nijhuis, W. F. Reus, J. R. Barber, M. D. Dickey, G. M. Whitesides, *Nano Lett.* **2010**, 10, 3611.
[16] N. Nerngchamnong, L. Yuan, D. C. Qi, J. Li, D. Thompson, C. A. Nijhuis, *Nat. Nanotechnol.* **2013**, 8, 113.
[17] H. J. Yoon, K. C. Liao, M. R. Lockett, S. W. Kwok, M. Baghbanzadeh, G. M. Whitesides, *J. Am. Chem. Soc.* **2014**, 136, 17155.
[18] L. Jiang, L. Yuan, L. Cao, C. A. Nijhuis, *J. Am. Chem. Soc.* **2014**, 136, 1982.

[19] L. Yuan, R. Breuer, L. Jiang, M. Schmittel, C. A. Nijhuis, *Nano Lett.* **2015**, 15, 5506.

[20] L. Yuan, N. Nerngchamnong, L. Cao, H. Hamoudi, E. del Barco, M. Roemer, R. K. Sriramula, D. Thompson, C. A. Nijhuis, *Nat. Commun.* **2015**, 6, 6324.

[21] A. Wan, C. S. Suchand Sangeeth, L. Wang, L. Yuan, L. Jiang, C. A. Nijhuis, *Nanoscale* **2015**, 7, 19547.

[22] A. R. Garrigues, L. Yuan, L. Wang, E. R. Mucciolo, D. Thompon, E. Del Barco, C. A. Nijhuis, *Sci. Rep.* **2016**, 6, 26517.

[23] P. Song, L. Yuan, M. Roemer, L. Jiang, C. A. Nijhuis, *J. Am. Chem. Soc.* **2016**, 138, 5769.

[24] G. D. Kong, H. J. Yoon, *J. Electrochem. Soc.* **2016**, 163, G115.

[25] G. D. Kong, M. Kim, S. J. Cho, H. J. Yoon, *Angew. Chem. Int. Ed.* **2016**, 55, 10307.

[26] Z. A. Lamport, A. D. Broadnax, D. Harrison, K. J. Barth, L. Mendenhall, C. T. Hamilton, M. Guthold, T. Thonhauser, M. E. Welker, O. D. Jurchescu, *Sci. Rep.* **2016**, 6, 38092.

[27] X. Chen, M. Roemer, L. Yuan, W. Du, D. Thompson, E. Del Barco, C. A. Nijhuis, *Nat. Nanotechnol.* **2017**, 12, 797.

[28] L. Qiu, Y. Zhang, T. L. Krijger, X. Qiu, P. V. Hof, J. C. Hummelen, R. C. Chiechi, *Chem. Sci.* **2017**, 8, 2365.

[29] M. Souto, L. Yuan, D. C. Morales, L. Jiang, I. Ratera, C. A. Nijhuis, J. Veciana, *J. Am. Chem. Soc.* **2017**, 139, 4262.

[30] H. Alon, R. Garrick, S. P. Pujari, T. Toledano, O. Sinai, N. Kedem, T. Bendikov, J.

E. Baio, T. Weidner, H. Zuilhof, D. Cahen, L. Kronik, C. N. Sukenik, A. Vilan, *J. Phys. Chem. C* **2018**, 122, 3312.

[31] A. D. Broadnax, Z. A. Lamport, B. Scharmann, O. D. Jurchescu, M. E. Welker, *J. Organomet. Chem.* **2018**, 856, 23.

[32] P. Song, S. Guerin, S. J. R. Tan, H. V. Annadata, X. Yu, M. Scully, Y. M. Han, M. Roemer, K. P. Loh, D. Thompson, C. A. Nijhuis, *Adv. Mater.* **2018**, 30, 1706322.

[33] G. D. Kong, J. Jin, M. Thuo, H. Song, J. F. Joung, S. Park, H. J. Yoon, *J. Am. Chem. Soc.* **2018**, 140, 12303.

[34] M. Baghbanzadeh, L. Belding, L. Yuan, J. Park, M. H. Al-Sayah, C. M. Bowers, G. M. Whitesides, *J. Am. Chem. Soc.* **2019**, 141, 8969.

[35] S. J. Cho, G. D. Kong, S. Park, J. Park, S. E. Byeon, T. Kim, H. J. Yoon, *Nano Lett.* **2019**, 19, 545.

[36] Z. A. Lamport, A. D. Broadnax, B. Scharmann, R. W. Bradford, 3rd, A. DelaCourt, N. Meyer, H. Li, S. M. Geyer, T. Thonhauser, M. E. Welker, O. D. Jurchescu, *ACS Appl. Mater. Interfaces* **2019**, 11, 18564.

[37] Y. Han, C. Nickle, Z. Zhang, H. Astier, T. J. Duffin, D. Qi, Z. Wang, E. Del Barco, D. Thompson, C. A. Nijhuis, *Nat. Mater.* **2020**, 19, 843.

[38] X. Qiu, V. Ivasyshyn, L. Qiu, M. Enache, J. Dong, S. Rousseva, G. Portale, M. Stohr, J. C. Hummelen, R. C. Chiechi, *Nat. Mater.* **2020**, 19, 330.

[39] H. Kang, G. D. Kong, S. E. Byeon, S. Yang, J. W. Kim, H. J. Yoon, *J. Phys. Chem. Lett.* **2020**, 11, 8597.

[40] J. Park, L. Belding, L. Yuan, M. P. S. Mousavi, S. E. Root, H. J. Yoon, G. M.

Whitesides, *J. Am. Chem. Soc.* **2021**, 143, 2156.

[41] A. G. C. Zhou, L. X. Zhu, H. Z. Yu, *J. Phys. Chem. C* **2022**, 126, 7638.

[42] X. Qiu, S. Rousseva, G. Ye, J. C. Hummelen, R. C. Chiechi, *Adv. Mater.* **2021**, 33, 2006109.

[43] G. D. Kong, H. Song, S. Yoon, H. Kang, R. Chang, H. J. Yoon, *Nano Lett.* **2021**, 21, 3162.

[44] N. Matsuhisa, S. Niu, S. J. K. O'Neill, J. Kang, Y. Ochiai, T. Katsumata, H. C. Wu, M. Ashizawa, G. N. Wang, D. Zhong, X. Wang, X. Gong, R. Ning, H. Gong, I. You, Y. Zheng, Z. Zhang, J. B. Tok, X. Chen, Z. Bao, *Nature* **2021**, 600, 246.

[45] L. P. Ma, J. Ouyang, Y. Yang, *Appl. Phys. Lett.* **2004**, 84, 4786.

[46] S. Steudel, K. Myny, V. Arkhipov, C. Deibel, S. De Vusser, J. Genoe, P. Heremans, *Nat. Mater.* **2005**, 4, 597.

[47] Y. Ai, S. Gowrisanker, H. Jia, I. Trachtenberg, E. Vogel, R. M. Wallace, B. E. Gnade, R. Barnett, H. Stiegler, H. Edwards, *Appl. Phys. Lett.* **2007**, 90, 262105.

[48] B. N. Pal, J. Sun, B. J. Jung, E. Choi, A. G. Andreou, H. E. Katz, *Adv. Mater.* **2008**, 20, 1023.

[49] S. Steudel, K. Myny, P. Vicca, D. Cheyns, J. Genoe, P. Heremans, *IEEE* **2008**, 93.

[50] J. Sun, B. N. Pal, B. J. Jung, H. E. Katz, *Org. Electron.* **2009**, 10, 1.

[51] D. Im, H. Moon, M. Shin, J. Kim, S. Yoo, *Adv. Mater.* **2011**, 23, 644.

[52] G. Gutierrez-Heredia, V. H. Martinez-Landeros, F. S. Aguirre-Tostado, P. Shah, B. E. Gnade, M. Sotelo-Lerma, M. A. Quevedo-Lopez, *Semicond. Sci. Technol.* **2012**, 27, 085013.

[53] C. M. Kang, J. Wade, S. Yun, J. Lim, H. Cho, J. Roh, H. Lee, S. Nam, D. D. C. Bradley, J. S. Kim, C. Lee, *Adv. Electron. Mater.* **2016**, 2, 1500282.

[54] F. A. Viola, B. Brigante, P. Colpani, G. Dell'Erba, V. Mattoli, D. Natali, M. Caironi, *Adv. Mater.* **2020**, 32, e2002329.

[55] K. Ferchichi, S. Pecqueur, D. Guerin, R. Bourguiga, K. Lmimouni, *Electronic Materials* **2021**, 2, 445.

[56] Y. Kim, G. Kim, B. Ding, D. Jeong, I. Lee, S. Park, B. J. Kim, I. McCulloch, M. Heeney, M. H. Yoon, *Adv. Mater.* **2022**, 34, e2107355.

[57] H. Wang, J. H. Hsu, G. Yang, C. Yu, *Adv. Mater.* **2016**, 28, 9545.

[58] J. Semple, S. Rossbauer, C. H. Burgess, K. Zhao, L. K. Jagadamma, A. Amassian, M. A. McLachlan, T. D. Anthopoulos, *Small* **2016**, 12, 1993.

[59] J. Wang, Y. Wang, K. Li, X. Dai, L. Zhang, H. Wang, *Adv. Mater.* **2022**, 34, e2106624.
